# Polymeric and biological membranes for organ-on-a-chip devices

**DOI:** 10.1038/s41378-023-00579-z

**Published:** 2023-08-29

**Authors:** Kendra Corral-Nájera, Gaurav Chauhan, Sergio O. Serna-Saldívar, Sergio O. Martínez-Chapa, Mohammad Mahdi Aeinehvand

**Affiliations:** School of Engineering and Science, Tecnológico de Monterrey, Ave. Eugenio Garza Sada 2501, Monterrey, 64849 Mexico

**Keywords:** Microfluidics, Materials science

## Abstract

Membranes are fundamental elements within organ-on-a-chip (OOC) platforms, as they provide adherent cells with support, allow nutrients (and other relevant molecules) to permeate/exchange through membrane pores, and enable the delivery of mechanical or chemical stimuli. Through OOC platforms, physiological processes can be studied in vitro, whereas OOC membranes broaden knowledge of how mechanical and chemical cues affect cells and organs. OOCs with membranes are in vitro microfluidic models that are used to replace animal testing for various applications, such as drug discovery and disease modeling. In this review, the relevance of OOCs with membranes is discussed as well as their scaffold and actuation roles, properties (physical and material), and fabrication methods in different organ models. The purpose was to aid readers with membrane selection for the development of OOCs with specific applications in the fields of mechanistic, pathological, and drug testing studies. Mechanical stimulation from liquid flow and cyclic strain, as well as their effects on the cell’s increased physiological relevance (IPR), are described in the first section. The review also contains methods to fabricate synthetic and ECM (extracellular matrix) protein membranes, their characteristics (e.g., thickness and porosity, which can be adjusted depending on the application, as shown in the graphical abstract), and the biological materials used for their coatings. The discussion section joins and describes the roles of membranes for different research purposes and their advantages and challenges.

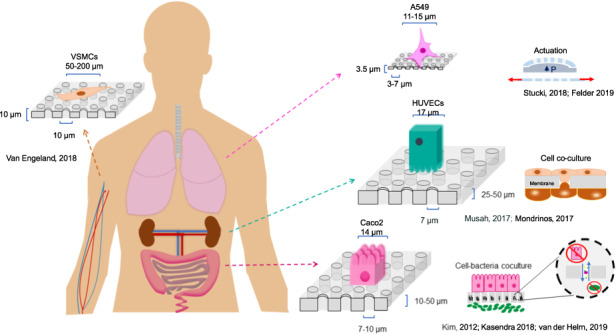

## Introduction

Organ-on-a-chip (OOC) membranes have gained relevance as a high-potential technology for research in tissue engineering^1^, drug discovery^2^, disease modeling, and precision medicine^3^. OOCs can mimic an organ function in a controlled in vitro environment, and membranes have helped broaden knowledge of physiological processes within a tissue. For example, OOCs enable cell differentiation; thus, the state at which a cell exhibits a unique morphology and/or functions more closely resemble that observed in vivo. Ranging from systems that implement a single cell line and fluid flow to the most complex multilayer tissue model, these miniaturized systems have been used to tackle biological questions regarding the effects of mechanical and chemical cues in tissue assembly and cellular interactions. OOCs offer a flexible alternative that rapidly evolves and should refine, reduce, and eventually replace animal testing by overcoming its disadvantages, including high costs, ethical concerns, and lack of representativity. Importantly, these devices have enabled studies, such as wound induction and repair in internal organs, that are otherwise extremely challenging and in some cases impossible to carry out in vivo given the inaccessibility of tissues^4^.

In the context of cell culture, OOC membranes are free-standing semipermeable thin films with pores (often smaller than cells) that allow drugs, metabolites, and nutrients to be selectively transported and are involved in cell communication and medium perfusion^5^; OOC membranes function as scaffolding (cell support), provide space separators, and sometimes perform actuation through valving or stretch contraction. Membranes, however, are not limited to film shapes, as the separatory and permeation functions can be supplied by an ECM cylinder^6^ or micropillar arrays^7,8^. For the purposes of this review, materials with interconnected micro- or nanopores that function as scaffolds, supports or substrates for cell culture will be referred to as membranes. Although a membrane acts as a barrier, it is very important to distinguish this separatory function in OOCs from that of filtration for other applications, such as wastewater treatment or applications in food and beverage industries^9^.

First, membranes function as a support for cell culture by acting as scaffolds. For this to happen, some degree of adhesion must be achieved. Cell-scaffold interactions and cell adhesion depend on several parameters, including membrane material, surface chemistry, and topography^10–13^. The ECM’s topography should be closely mimicked, as it influences behavior such as migration and specialization^11,14^. Most synthetic membranes do not effectively mimic the native ECM properties and require protein coatings (collagen, elastin, fibronectin, or laminin) to enhance cell adhesion^15,16^. Growth factors, such as vascular endothelial growth factors (VEGF), can also be incorporated into the scaffold to promote cell survival^17^. Nevertheless, it is important to note that the microenvironment (e.g., media and gas supply, scaffold composition, etc.) also stimulates the receptors expressed by the cells (e.g., epithelial cells bind to type IV collagen), and thus the binding of the adherent cells to the ECM or the membrane^18^.

The separatory role of membranes involves maintaining cells and fluids in a chamber while allowing substances to migrate and exchange through the pores^19^. Different cells can be seeded on both sides of a membrane or even migrate across it, depending on the membrane’s thickness and the pore features, which enable cell communication and cell‒cell contact^20^. Pore size is crucial for studying mechanisms, such as the mechanisms behind the immune response, homeostasis, cellular growth, shape, absorption/excretion, and communication^14^. When selecting a membrane, other factors that should be considered include optical transparency for microscopy imaging^19^ and tensile properties^21^ for the mechanical stimulation of cell layers. For example, in organs that require cyclic mechanical stimulation, such as the lung and intestine, flexible porous membranes can aid in mimicking breathing or peristaltic motions, respectively^3^. Furthermore, the adjustment in physiological flows that generate shear stress over the cells is a fundamental parameter for their polarization and subsequent differentiation observed in vivo^20^. The types, applications, and characteristics of different membranes used in OOCs, which are discussed in the manuscript, are summarized in Table 1 for the reader’s convenience.

**Table 1:** Membranes in OOC studies, characteristics, and applications

Membrane material	Application study	Fluidic flow/shear stress	Stimuli	Fabrication method/provider, bonding	Thickness	Porosity diameter; center-to-center/density	Biological coating
PDMS	Gut: Recapitulation of intestine Coculture with *L. rhamnosus*^41^	30 mL/h 0.02 dyne/cm^2^	Cyclic strain (10%; 0.15 Hz)	Soft lithography (casting on a microfabricated silicon wafer from MEMS and Nanotechnology Exchange) Oxygen plasma	30 μm	10 µm; 25 µm	Matrigel® and collagen Type I (Caco-2)
PDMS	Small intestine: Biopsy-derived epithelium cultured on-chip for transcriptomic comparison; duodenum-like^118^	60 μl/h	Strain (10%; 0.2 Hz)	Both fabricated (Kim 2012 soft lithography) & purchased from Emulate, Inc. Oxygen plasma	50 μm	7 μm; 40 μm	Matrigel® and collagen Type I (HIMECs)
PDMS	Gut: Transepithelial barrier structure and tissue structure (impedance spectroscopy)^117^	30 μl/hr. 4.1 × 10^−4^ dyne/cm^2^ (41 μPa)	Electrical stimulation AC current of 10 μA at 50 frequencies in the range of 100–10 Hz	Kim 2012–soft lithography (casting on a microfabricated silicon wafer) Oxygen plasma	50 μm	7 μm; 40 μm	Matrigel® and collagen Type I (Caco-2)
PDMS	Intestine: Host-microbiome interactions^33^	60 μl/hr.	N/A	Huh 2013 - silicon wafer using photolithography, removing silicon by DRIE. Obtained from MEMS and Nanotechnology Exchange Organ Chip (Emulate Inc.) Oxygen plasma	10 μm	10 µm; 25 µm	Matrigel® and collagen Type I (Caco-2, HIMECs)
PDMS	Kidney: coculture of primary glomerular endothelial and hiPS-derived podocyte Recapitulation of glomerulus, selective permeability^43^	60 μL/h Top channel: 0.0007 dyne/cm^2^ Bottom channel: 0.017 dyne/cm^2^	Cyclic strain (10%; 1 Hz, −85 kPa)	Huh, 2010. Stereolithography molds (ProtoLabs) and spincoating Oxygen plasma	50 μm	7 μm; 40 μm	Laminin (hiPS-derived podocytes)
PDMS	Alveolus: Thrombosis model Primary human lung alveolar epithelial cells culture, human whole blood used. Evaluation of therapeutic alternative: protease activated receptor-1 (PAR-1) antagonist.^24^	Wall shear rate ~10 dyne/cm^2^ (250 s^−1^)	N/A	Casting against DRIE silicon wafer Oxygen plasma	50 μm	7 μm; 40 μm	Collagen I, fibronectin (Primary alveolar cells, HUVECs)
PDMS	Gut: Independent control of fluid flow and mechanical deformations to explore the influence of each in morphogenesis^34^	30 mL/h	Cyclic strain (10%; 0.15 Hz)	Kim 2012–soft lithography (casting on a microfabricated silicon wafer) Oxygen plasma	20 μm	10 µm; 25 µm	Matrigel® and collagen Type I (Caco-2)
PDMS	Alveolus: Replication of breathing motion, air‒blood barrier and air-liquid interface Coculture: primary human lung alveolar cells (hAEpCs) and primary lung endothelial cells^48^	N/A	Cyclic strain (0.2 Hz)	Microstructuring-lamination Oxygen plasma	3.5 μm	3 µm; 800,000 pores/cm^2^	Collagen IV (epithelial cell line, 16HBE14o), collagen I (primary cell line, hAEpC)
PDMS	Lung: Replication of idiopathic pulmonary fibrosis (IPF), pathological study on wound healing Treatment with recombinant human hepatic growth factor (rhHGF)^4^	N/A	Cyclic strain (10%; 0.2 Hz)	Microstructuring-lamination Clamping (sandwich)	3.5 μm	3 µm; 800,000 pores/cm^2^	Fibronectin (A549 epithelial cells)
PDMS	Vessel: Coculture of aortic endothelial cells (ECs) and human aortic vascular smooth muscle cells (VSMCs) Mimicking of arterial wall^44^	20 μl/min shear stress of 1–1.5 Pa and strain of 5–8%	Pressure flow: 1 mbar/h increments until 15 mbar ( ≈ 1–1.5 Pa endothelial shear stress) Vacuum pressure −10 mbar/hr. steps until −200 mbar	Laser excimer (UV) Oxygen plasma (asher)	10 μm	10 µm; 28 µm	Fibronectin (ECs- HaVECs, VSMCs- hAoSMCs)
PDMS	Microfluidic device: Combine temperature, pressure, and moisture to generate micron-scale pores on a PDMS membrane. Relate those parameters with pore size and adhesion to DNA, and collagen.^79^	20 μl/min	N/A	High-pressure saturated steam Thermal treatment	40 μm	5 µm	Parylene-C, collagen (platelets)
PDMS	Organ-on-a-chip: Viability of cell culturing: Human umbilical endothelial cells (HUVEC) and MDA-MB-231 (MDA) cells^14^	N/A	N/A	Photolithography, dry and wet etching PDMS/toluene mortar; Oxygen plasma	1–4 μm	2–10 μm; 8–65%	Fibronectin (HUVEC-primary, MDA-cancer)
SiO_2_	Chip: Ultrathin membrane for support of physiologically relevant cellular interactions Optically transparent Human umbilical vein endothelial cells (HUVECs) spread and proliferate on these membranes.^69^	N/A	N/A	Photolithography and reactive ion etching Ozone	300 nm (comparable in thickness to the vascular basement membrane of 100–300 nm)	0.5 and 3 μm; 27.5%	Geltrex (Life Technologies, California) HUVECs
PLGA	Lung tumor: Gefitinib drug testing Permeability, coculture^1^	N/A	N/A	Electrospinning PLGA direct sealing	3 μm	NA, nanofiber	(A549 epithelial cells)
Collagen-elastin	Lung: Air‒blood barrier replication, exposed to mechanical forces Gold mesh to mimic alveolar size and structure^3^	N/A	Strain 10%; 4.0 kPa	Gelation Double tape	10 μm		hAEpCs (primary cells)
Collagen	Kidney: Endothelial-epithelial exchange interface, reabsorption mechanism^137^	10 µl/min Wall shear stress ~1–10 dyne/cm^2^	N/A	Compression molding Screwing	25 μm	NA	hRVTU, HUVECs
Collagen	Colon: Suitability of membrane for OOC, comparison w/Transwell ® Microstructure, transport and cell viability^136^	N/A, media changed daily	N/A	Lyophilization Sandwiching	~15 μm	~10.2 μm	Caco-2
Collagen	Microfluidic device: Cell attachment, growth physiologically relevant in vitro cell culture models^19^	70–100 µl/hr.	N/A	Vitrification PDMS mortar	20 μm	250 nm	HUVECs
PDMS*	Colon: Mucus layer physiology^119^	60 µl/hour	N/A	CHIP-S1 Stretchable Chip, RE00001024 Basic Research 1680 Kit; Emulate, Inc	50 μm	7 μm; 40 μm	Matrigel® and collagen Type I (primary colonic intestinal epithelial cells)
PDMS*	Spinal cord: Vascular-neural interaction Specific gene activation enhanced neuronal function and in vivo-like signatures.^102^	N/A	N/A	Emulate, Inc.	50 μm	7 μm; 40 μm	Matrigel® (neural, spMNs) Collagen IV and fibronectin (vascular, BMECs)
Polyester*	Intestine: Long-term culture up to 30 days Rhohdamine 123 basal to apical flow^103^	N/A	Magnetic stir bar to pump media	Corning (Transwell ®) Oxygen plasma	10 μm	0.4 μm	Type I collagen (Caco-2)
Polyester*	Kidney: Primary rat inner medullary collecting duct (IMCD) cells^115^ Primary kidney epithelial cells Enhanced epithelial cell polarization and primary cilia formation. Cisplatin toxicity and Pgp efflux transporter activity measured on-chip.^110^	0.2 dyne/cm^2^ (physiological conditions renal system: 0.2–20 dyne/cm^2^, ~10% of the endothelial cell) 1 dyne/cm^2^ for 5 h	N/A	Corning (Transwell ®) Oxygen plasma	10 μm	0.4 μm	Fibronectin, Collagen Type IV (PTEpiC, primary)
PET*	Gut: Immune response, microbial pathogenicity mechanisms, and quantification of cellular dysfunction Probiotic *L. rhamnosus* Pathogen *Candida albicans*^161^	Flow rate: 50 μl/min Endothelial: 0.07 Pa Luminal: 0.01 Pa	N/A	TRAKETCH Sabeu, Radeberg, Germany Polystyrol (PS) foil (microfluidic ChipShop, Germany)	12 μm	8 μm; 1 × 10^5^ pores/cm^2^	HUVECs, Caco-2
PET*	Gut: Transport study of amoxicillin, antipyrine, ketoprofen and digoxin^27^	100 μL/h	N/A	N/A Clamping	12 μm	0.4 μm; 1.6 × 10^6^ pore density	Caco-2
PET*	Small intestine-Liver: Caco-2, HepG2, and A549 cell cultures were used as organ models of the small intestine, liver, and lung, respectively. Pharmacokinetics: —epirubicine (EPI), irinotecan (CPT-11), and cyclophosphamide (CPA)^104^	Rotation frequency of 1600 rpm to generate a flow rate of 0.16 µL/s	N/A	N/A Oxygen plasma	--	--	Collagen (Caco-2, HepG2)
PET*	Heart: Centrifugally assisted cell loading^50^	Flow rate: 50 µL/h	Centrifugation (3 min at 138 · g)	SABEU 030444 Oxygen plasma	3 μm	--	Fibronectin (hiPSC-derived CMs)
Teflon*	Gut: Electrodes for transepithelial electrical resistance (TEER) measurements for real-time monitoring of barrier integrity Permeability studies to evaluate differentiation. Mucus production^116^	Day 1 flow rate of 0.5 μl/min, upper layer Day 2 onward 3 μl/min, both chambers, shear stress ~ 0.008 dyne/cm^2^ at (Physiological conditions epithelial cells: 1 to 5 dyne/cm^2^)	N/A	Millipore, Denmark UV radiation	40 μm	0.4 μm; ~ 75%	Matrigel® and collagen Type I (Caco-2)
PC*	Lung: 3D culture-specific-morphology, maintained excellent barrier integrity, secreted mucus, and expressed cell surface functional P-glycoprotein Effects of cigarette smoke extract (CSE) on Interleukin-6 (IL-6) and Interleukin-8 (IL-8) release^90^	30 μl/h	N/A	Merck, Germany Oxygen plasma	10 μm	0.4 μm; 15%	Matrigel® and collagen Type I (Calu-3, epithelial)
PC*	Blood Brain-Barrier: TEER measurements for any organ-on-chip device with two channels separated by a membrane.^162^	N/A	N/A	Corning (Transwell ®) PDMS/toluene mortar	10 μm	0.4 μm; 15%	Fibronectin (hCMEC/D3)
PC*	Intestine: Coculture of human and microbial cells under representative conditions *Lactobacillus rhamnosus GG, Bacteroides caccae*^108^	25 ml/min	N/A	GE Healthcare Gasketing	NS	1 μm;	Collagen, fibronectin (Caco-2; BeWo trophoblast)
PC*	Placenta: Screen drug compounds for their ability to cross the placenta Heparin, glyburide^40^	Flow rate 100 µl/h.	N/A	GE Healthcare PDMS mortar	NS	1 μm;	Fibronectin (HPVEC)

Membranes without * sign are been fabricated by the authors, and those with * sign are commercially available and have been purchased for the intended studies

A recent work by Rahimnejad et al. provides a thorough overview of the desirable characteristics of membranes and their significance in OOC devices^22^. The paper reviews the most relevant organs and the milestones that have been achieved in mimicking physiological features with microfluidic devices. In contrast, our review focuses on the relevance of mechanical conditions in OOCs and the membrane aspects that influence physiological representativity. In addition, methods and materials used to fabricate membranes are discussed. We provide specific examples that describe the material and porosity of the membrane, as well as the impact of its characteristics on the results obtained. This includes the use of biological coatings to increase physiological representativity. Our review also highlights that the previous domination of PDMS in microfluidics may face a decline as more biological membranes are being explored.

## Relevant mechanical conditions for increasing the physiological relevance of cells

**Fig. 1:**
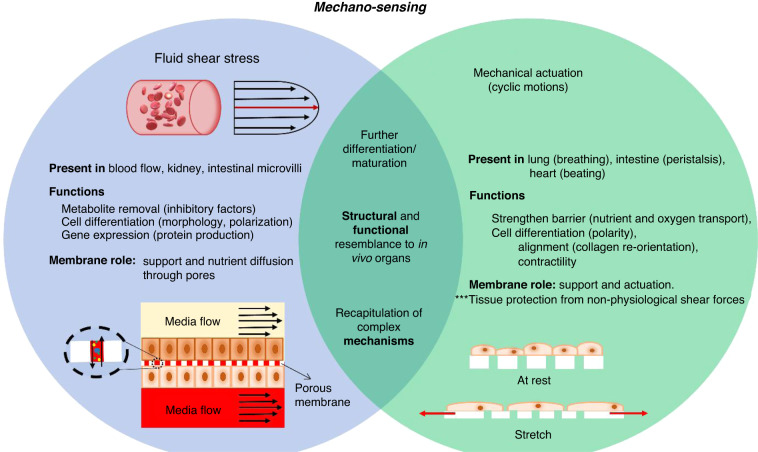
Membranes mediate cell mechanosensing under fluid shear stress and mechanical actuation. The combination of fluid shear stress and mechanical actuation results in an increased cell differentiation, achieves a closer resemblance to in vivo organs and allows the recapitulation of complex biological mechanisms, as opposed to the mechanical stimulation methods individually

Shear stress and chemical and mechanical cues are not only crucial for cell survival but also impact cellular activity, such as cell migration, mass transport via ion channels, and protein conformational changes^23^. As shown in Fig. 1, the implementation and combination of physiological flows and mechanical stress in OOCs are essential for elucidating cell interactions and responses to their microenvironment^11^. This has become increasingly relevant for pathological studies since more realistic physiological environments (e.g., whole blood perfusion) enable more physiological responses (e.g., patient-derived cell culture)^24^. In these regards, membranes on OOCs indirectly modulate cell exposure to external forces (shear stress, fluid flow, mechanical stimulation) by acting as supports, barriers, or shock absorbers. This section reviews studies conducted on OOCs with polymeric membranes in which the effects of fluidic shear stress and mechanical actuation in different tissue cultures are discussed.

### Fluid shear stress in OOCs

Fluid shear stress (FSS) is immediately present in the body through blood flow, playing a key role in metabolite removal and triggering phenotypic changes^25^. As FSS influences cellular structure and function, it is a key factor and should be considered when creating in vitro microenvironments that closely mimic those cells experience in vivo^26^. In many OOCs, continuous fluid flow removes metabolites, limiting the interaction with cells and uptake^27^. Although detrimental to drug exposure studies, this feature leads to the removal of inhibitory factors, which aids the cell’s increased physiological relevance (IPR), gene expression, and cellular performance. In turn, cell IPR (e.g., through tight junctions that are required for barrier function) helps restrict substance permeability in mature cells.

Notably, porous membranes affect flow within OOC devices. A study by Chung et al. calculated the permeability and distribution of flow rates based on the thickness and porosity of the membrane^28^. Their findings showed that characterizing the permeability of a tissue barrier in terms of solute diffusion, fluid flow, and electric current can guide the design and evaluation of tissue barrier and coculture models. Solute permeability is an indicator of tissue barrier tightness and active transport rate and can be estimated via the permeability of tracer molecules. Fluid permeability, on the other hand, indicates mature barrier formation, and the general leakiness of a tissue barrier can be quickly assessed through transepithelial/transendothelial electrical resistance (TEER). FSS thus creates a dynamic microenvironment within OOCs, interacting with elements such as a porous membrane, that brings cells a step closer to organ-level structure and function.

### Creating a dynamic environment to promote cell specialization

**Fig. 2:**
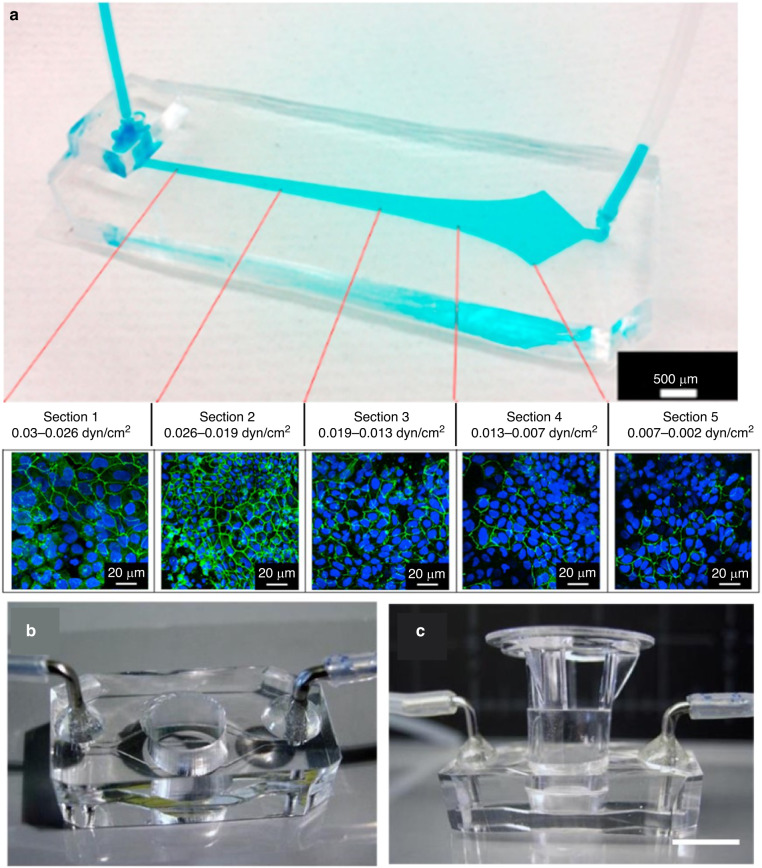
**Fluidic flow relevance in microfluidic devices.** **a** OOC developed by Delon et al. (2019) to investigate the effect of fluid shear stress on Caco-2 intestinal epithelial cells. The device is sectioned to deliver differential shear stress (top), with the highest shear stress on the left and the lowest, on the right (Image adapted from Figs. 1 and 6 from reference^32^). The formation of tight junctions is monitored via immunofluorescence staining (bottom) of ZO-1, a tight junction protein stained in green. **b**, **c** Hybrid insertable fluidic devices have been proposed by Shin et al. (2019) to introduce fluid flow to Transwell ® inserts (Images adapted from Figure S5 from reference^34^ Supplemental Information)

The relevance of shear stress can be traced to its effect on cellular machinery, which senses and responds to external changes. For example, brain microvascular endothelial cells respond to FSS (e.g., capillary-like shear stress ~6 dynes/cm^2^) by increasing cytoskeleton protein production as well as tight junctions and transporter proteins^29^. In the kidney, FSS (e.g., 0.2–5 dynes/cm^2^) contributes to F-actin polymerization and depolymerization, which impacts renal cell tubular morphology and polarization^30,31^. Microvilli formation and mechanotransduction in intestinal epithelial cells, such as Caco-2 cells, are influenced by FSS (e.g., 0.002–0.03 dynes/cm^2^). Shear stress causes epithelial brush borders to be reorganized, which is fundamental for intestinal function, through stimulating the mechanosensing proteins F-actin (a cytoskeletal protein) and villin (an actin-binding marker of intestinal differentiation)^32^. As shown in Fig. 2a, Caco-2 cells’ tight junctions formed at medium FSS (between 0.02 and 0.01 dyn/cm^2^) but decreased at low (0.002 dyn/cm^2^) and high (0.03 dyn/cm^2^) FSS. High FSS levels increase vacuolization (a cellular stress indicator), the production of protective mucus, and mitochondrial activity due to the dependence of barrier integrity on ATP^32,33^.

FSS, along with intra- and extracellular stimuli, impacts cell specialization and eventually enables FSS sensing to create a feedback loop. In addition, drugs, metabolites, and other substances can be removed through FSS. As the following cases will demonstrate, these functions are not mutually exclusive.

### Using FSS to study cell differentiation mechanisms in vitro

When using OOCs in which cells are exposed to FSS, a membrane is usually implemented to provide support and achieve nutrient diffusion through the pores. Shin et al. conducted a study to elucidate the effect of basal and apical fluid shear stress on epithelial cells cultured in a gut-on-a-chip. The device consisted of two apposed microchannels separated by an ECM-coated PDMS porous membrane (thickness 20 μm, pores 10 μm diameter with 25 μm spacing). The authors demonstrated that applying FSS as low as 0.02 dyn/cm^2^ simultaneously to apical (top) and basal (bottom) channels achieved 3D morphogenesis, but when the flow was applied only on the bottom chamber, differentiation took 1.5 times longer. Apical shear stress was insufficient to induce differentiation in a single-channeled device, in which a monolayer with occasional epithelial domes formed. This result suggested that intestinal epithelial cells, when polarized, might secrete an inhibitory factor that concentrates over the basal membrane and inhibits the differentiation of epithelial cells to villi^34^. To further test this hypothesis, researchers collected medium from Caco-2 cells cultured in a static Transwell for three days and flowed the samples through the bottom chamber of the gut-on-a-chip while maintaining continuous fluid flow on the apical chamber. This caused the 3D morphogenesis of Caco-2 cells cultured in the gut-on-a-chip to stop. Furthermore, the authors transferred a three-week Caco-2 monolayer (grown in a static Transwell ® setup) into a hybrid fluidic device (Fig. 2b, c) created from silicone. Fluid flow was applied to the bottom chamber alone, and within 48 h, 3D morphogenesis was observed^34^.

The effect of shear stress on intestinal cell differentiation is critical because it results in increased absorption, mucus formation, and other intracellular metabolic activities^35^. Blood flow, lymphatic flow, and other types of bodily fluid movement contribute to the production of FSS in vivo. Leung et al. further summarized that over a certain optimal range, physiologically experienced FSS displays direct proportionality with the phenotypes and functionality of differentiated cells^36^. Several examples using OOC models presented the significance of FSS on cell differentiation, especially in tissues such as the endothelium, bone, and cartilage that undergo substantial shear stress. In a study by Lembong et al., a microfluidic platform was designed to finely control the medium flow and fluid shear stress. The system was used to dynamically culture human mesenchymal stem cells (hMSCs) and quantify their osteogenic differentiation^37^. The microfluidic chambers consisted of vertical cylindrical pillars (pillar diameter: 1 mm, pillar-to-pillar distance: 2.5 mm), and cells were seeded within ∼200 μm. The study reported a 10-fold increase in the expression of the osteogenic marker alkaline phosphatase (ALP) due to flow-induced shear stress. In another study by Trieu et al., a microfluidic device that uses airflow to polarize ciliated airway epithelium was developed^38^. This study aimed to evaluate the effect of FSS on the polarization and differentiation of airway epithelial cells by simulating the in vivo environment of the respiratory tract. A bronchial epithelial cell line (BEAS2B) and primary human tracheal epithelial cells (HTECs) were seeded on a commercial polyester porous membrane (Costar Transwell 0.4 μm diameter) coated with collagen and fibronectin. The system successfully exposed cells to physiologically similar shear stress and induced the alignment and polarization of ciliated cells. After 24 hours of airflow exposure, the viability of both cell lines within the device was revealed, along with normal differential cilia development.

Another study by Faley et al. investigated the role of shear stress in the stabilization and enhancement of barrier integrity in human brain microvascular endothelial cells (BMECs) derived from induced pluripotent stem cells (iPSCs)^39^. The BMECs were cultured on gelatin hydrogel (approximate thickness 60 μm) under continuous perfusion in a channel coated with collagen IV and fibronectin. The results showed that barrier function declined on Day 7 for static culture, and the values for continuous perfusion culture remained similar to the initial values after 2 weeks. Nonperfused cells also showed an increase in permeability. The authors also explored stop-flow conditions, which allowed nutrient exchange with minimal exposure to shear stress. Under these conditions, the cells showed similar or greater permeability than that of static cultures. The authors concluded that although shear stress is not a determining factor for BMECs to establish tight junctions, it may provide a positive contribution to the barrier function and integrity, probably through mechanical stimulation and the reduction of oxygen-species degradation^39^.

### Utilizing FSS to more closely mimic organ-level functions

In another study by Blundell et al., the authors investigated glyburide (a gestational diabetes drug) transport and cell differentiation in a placenta-on-a-chip with two channels separated by a fibronectin-coated semipermeable polycarbonate membrane (1 μm diameter pores)^40^. As a placental barrier, the membrane was seeded with trophoblast and human placental villous endothelial cell monolayers on opposite sides. The study showed that continuous perfusion triggered microvillus formation in the placental barrier due to increased proliferation and intercellular junction formation. The differentiation of the placental barrier successfully limited fetal drug exposure as it mimicked the transport mechanism of the breast cancer resistance protein (BCRP) that pumps components from the fetal compartment (basal or bottom chamber in the device) back to the maternal circulation. In these studies, multilayered organs were mimicked and the cell layers on opposing sides of the membranes differentiated through simultaneous contact with fluid flow or FSS. In OOCs, cell exposure to external mechanical cues, such as FSS and stress/strain, can be adjusted to generate distinct specialization profiles, while membranes could enable communication between chambers and cell layers and provide support, further nurturing the dynamic enclosed microenvironment.

## Membrane mechanical actuation

**Fig. 3:**
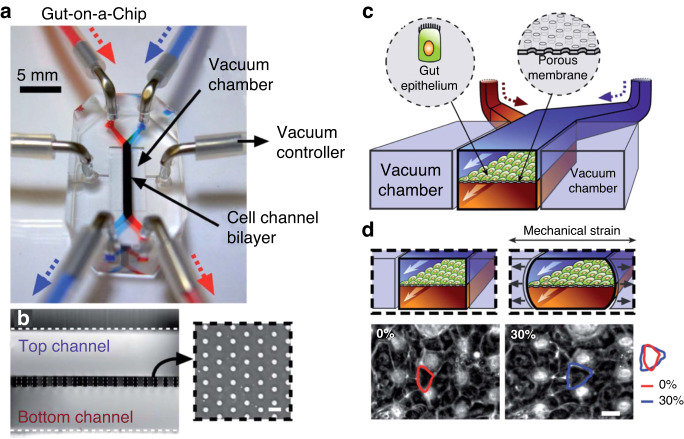
**OOC mechanical actuation.** **a** A gut-on-a-chip proposed by Kim et al. (2012). **b**, **c** The device has a basal‒apical conformation, channels are separated by a porous PDMS membrane. **d** The lateral vacuum chambers stretch the membrane, delivering mechanical stimulus to cells to mimic peristaltic motions. Image adapted from Fig. 1 from reference^41^

Mechanical stimulation of the membrane via cyclic motions is physiologically relevant for lung cells that undergo breathing, intestinal cells that experience peristaltic motions, or heart cells during beating. Mechanical stimulation is usually delivered via vacuum suction of a pair of lateral chambers on both sides of a membrane^34,41–44^ (Fig. 3) or through electrically generated negative pressures^3,4^.

The application of mechanical stimuli to in vitro biological systems broadens knowledge of normal and pathological processes. For example, transport phenomena and host-microbiome studies have demonstrated that the peristaltic motions of the intestinal epithelium affect the transport of oxygen and nutrients, as well as gut microbiota and mucosal homeostasis^45,46^. In another instance, cardiac tissues subjected to uniaxial stress in vitro present higher cell viability, IPR, and contractility^47^ due to the formation of junction complexes that propagate electrical stimuli for synchronous beating. In the lung, breathing motion influences epithelial polarity and IPR. This is fundamental to culture stress-sensitive cells, such as patient-derived primary alveolar cells (that normally cannot proliferate in vitro), which was achieved by Stucki and collaborators using micro diaphragm deflection via cyclic vacuum (0.2 Hz and 8% strain) on a 3.5 μm-thick PDMS membrane with 3 μm diameter pores^48^. Another study involving alveolar tissue demonstrated that cyclic mechanical stretch can severely impair wound healing^4^. The study employed a similar membrane to investigate idiopathic pulmonary fibrosis (IPF, a disease in which scars present lower elasticity than healthy cells) and found that scarred areas experience higher mechanical stress due to the lack of elasticity. In comparison to static cultures, the increased strain caused by “breathing” mechanical motions significantly impairs wound healing and causes apoptosis^4^. Notably, the discontinuous support of the porous membrane, in this case, was detrimental since the cell-ECM interaction is critical to cell growth and repair^4^.

Mechanical actuation has also been shown to impact cell alignment, which may be driven by the strain avoidance mechanism or the presence or absence of a restraining boundary condition (stretch or restrain)^49^. Strain avoidance, or orientation perpendicular to the applied cyclic stretch, occurs in endothelial cells, fibroblasts, mesenchymal stem cells, and osteoblasts. Finally, in addition, to support and actuation, membranes in OOC may also protect the tissue from the adverse effects of nonphysiological shear forces; for example, when loading hiPSC (human induced pluripotent stem cell)-derived cardiomyocytes into a chip using centrifugation, the membrane confines convective transport in the medium module, while the cells located in the tissue chamber remain viable and functional^50^.

### FSS and mechanical actuation in OOCs

The combination of complex mechanical conditions, such as FSS and mechanical stimulation, leads to the generation of organ-level structures and functions that more closely resemble those observed in vivo, allowing the recapitulation of complex mechanisms^51^. For example, a kidney-on-a-chip that cocultured hiPSC-derived podocytes and human kidney glomerular endothelial cells under cyclic stretch by vacuum suction (1 Hz; 10% strain) and pulsed fluid flow (60 mL/h) successfully replicated the differential clearance (selective filtration) of the glomerular capillary wall^43^. The authors demonstrated that, in comparison to FSS alone, applying FSS and mechanical stimulation results in significantly higher (*p* < 0.0001) staining of nephrin, which is a protein indicator of podocyte differentiation. Although both forces contribute to podocyte maturation, mechanical stimulation was shown to be fundamental for functional glomerular filtration. Mechanical stimulation promotes cellular spread, native ECM protein deposition (e.g., collagen IV), and soluble factor production (e.g., VEGF or vascular endothelial growth factors) that modulate key signaling pathways, such as glomerular development and podocyte lineage determination^43^.

Another example is vessels, which undergo cyclical strain in vivo due to transmural pressure and wall shear stress from blood flow friction^52,53^. Transmural pressure causes vessel wall expansion (deformation in all directions) and alters shear stress, which plays a role in the blood flow direction. Both forces induce endothelial cell sprouting and increase barrier function^54^. A model proposed by Van Engeland and collaborators cultured human aortic endothelial cells (ECs) and human aortic vascular smooth muscle cells (VSMCs) under a fluid flow of 20 μL/min and cyclic strain by suction (5–8%)^44^. These cells not only sense and respond to alterations in blood flow but also intercommunicate to regulate the formation and remodeling of the vessel wall architecture^44^.

## Membrane characteristics

**Fig. 4:**
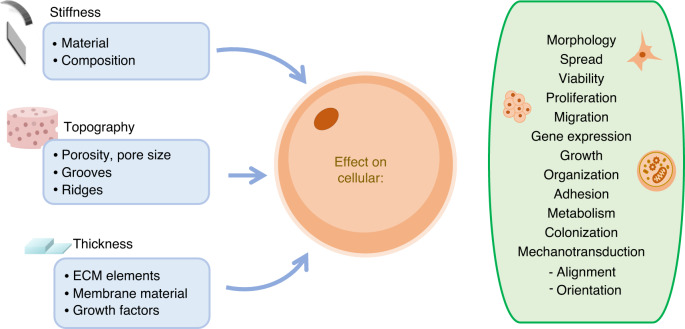
Membrane characteristics can be adjusted to mimic in vivo surfaces and promote cellular specialization. Stiffness, topography and thickness impact cellular structure, function and activity

Cells can sense changes in their environment, including mechanical and chemical changes/cues that influence their adhesion, morphology, growth, communication, and migration processes^55,56^. To achieve this sensitivity, membrane properties such as stiffness must be precisely controlled. Stiffness can be adjusted via composition variations, topography, and thickness, which can be achieved via micro- and nanofabrication^23^. The main parameters used to develop a membrane that functions as a scaffold for cell culture are shown in Fig. 4.

### Stiffness

Substrate stiffness generates differential patterns of gene expression related to the ECM and adhesion proteins, which impact cellular activity^56^. For example, when cultured on a stiff surface, human mesenchymal stem cells (hMSCs) are more spread, exhibit more stable focal adhesion, and present faster migration and higher proliferation rates^56^. These same cells have demonstrated that soft matrices that mimic the brain are neurogenic, while stiffer, muscle-like scaffolds are myogenic, and rigid collagenous substrates are osteogenic^11^. In this context, materials that are affected by the surrounding environment, such as dry collagen membranes that are rigid and resist bending when dry but are soft and gelatinous when wet^19^, become particularly relevant in hybrid microenvironments (e.g., lung) in which one chamber is exposed to media and the other is exposed to air.

### Topography

The membrane’s topography involves relief features such as porosity that can promote tissue adhesion and assembly^56^. Porosity, for example, allows cell migration and increases surface area^23^. The membrane’s surface relief structures can be positive or negative^57^ and influence contact guidance or cell orientation in response to geometries or fibers^58,59^. Positive topographies can be achieved via surface modification with nanospheres^60^, micropillars, or nanoparticles that provide anchor points^61^. Negative topographies include ridges and grooves^12,62,63^. Cells exposed to FSS present higher adhesion to membrane surfaces with groove topographies, as they resemble the native ECM^12^. Nanogrooves have been implemented to study the alignment of Duchenne muscular dystrophy (DMD) muscle cells on PDMS. A study conducted by Xu and collaborators showed that healthy cells align perpendicular to the grooves, while DMD-derived cells deviate from the structures^64^. This finding underscores the importance of the topography-responsive Dystrophin-Associated-Protein-Complex (DAPC), a protein complex that mediates the cytoskeleton-ECM interaction. DAPC enables perpendicular fiber alignment, and defects in the dystrophin protein, other DAPC components, or its interactions with laminin result in DMD^64^. Another example is bone tissue, as osteoprogenitors cultured on a nanogrooved polycaprolactone membrane presented an increase in cellular polarization and focal adhesions due to contact guidance^65^. The aligned mineralization of osteoblasts has also been reported in nanogrooved polystyrene, in which nanogrooves likely serve as nucleation points or templates for features as small as 50 nm in width and 17 nm in depth^66^.

### Thickness

Membrane thickness influences cell communication, contact, and even tissue structure^67^. Track-etched commercial membranes and many replica-molded PDMS membranes possess thicknesses of ~10 μm that hinder the needed protrusions and juxtapositions between cell types through the pores^44^. In vivo, alveolar membranes are as thin as 2.2 μm^48^, the placenta reaches ~4.53 μm at term^40^, and the vessel basal lamina is less than 100 nm thick^19^. Membrane thickness, however, cannot be reduced without compromising structural integrity, and this tradeoff must be carefully considered. Nonetheless, membranes thinner than 10 μm have been reported and will later be discussed in more detail. Other important considerations are the reproducibility and reliability of the fabrication method, as well as the environment to which the membrane will be exposed, e.g., the fluid flow pressure that could induce stress and deformation on the membrane.

## Fabrication of synthetic polymeric membranes

Commercially available polymer membranes are commonly used in OOC research^68^, and their characteristics vary across fabrication methods and materials. Polycarbonate (PC) and polyethylene terephthalate (PET) membranes are well-known and frequently implemented due to their wide availability and robustness^2,67^. These membranes are track-etched and possess very precise features. Nevertheless, they present limited transparency that hinders the optical characterization of cell cultures^69^ and high stiffness that makes them unsuitable for mechanical stimulation and stretching^14^. In addition, although the pore diameter is well controlled, placement is random, and thus, the porosity must be low to prevent the pores from overlapping. Moreover, their thickness is usually fixed at approximately 10 μm, which hinders cell‒cell (juxtacrine) signaling that requires cell-membrane-to-cell-membrane contact^67,70^. Another fabrication alternative is electrospinning, which cannot control pore size and location well but provides highly porous scaffolds (up to 80%) for cell culture, fully interconnected pores, and a high surface-to-volume ratio^71^. The similarity to native ECM and biocompatibility of electrospun synthetic polymers, such as poly-lactic acid (PLA) or poly(lactic-co-glycolic acid) (PLGA), have yielded this technology in areas such as bone tissue regeneration and drug delivery^72,73^. Electrospun fibers have also been successfully incorporated in on-chip devices; for example, the device designed by Yang et al. to mimic the lung for drug testing (3 μm thickness)^1^ or to entrap cancerous cells (8 μm thickness) as proposed by Xu et al.^74^. Important challenges concerning electrospun membranes include the need for conductive biocompatible polymers that can be electrospun^75^, the potential cytotoxicity of residual solvent in the fibers, their lack of transparency for microscopy imaging and the nonuniform cell distribution given the random alignment of the fibers, which limits vascularization^76^.

The lack of transparency and stiffness are effectively overcome by PDMS, a transparent silicone that has significantly overtaken microfluidic device fabrication due to its versatility^77^. PDMS has remained a relevant polymer for porous membrane fabrication because it exhibits several advantages, including accessibility, application tunability, and fabrication feasibility through soft lithography or replica molding. By utilizing photolithography or dry etching to create molds, the pore shape, location, and separation can be precisely controlled. In general, due to the establishment, replication, and refinement of OOC fabrication protocols, PDMS has become a selected material for routine OOC use^78^. Notably, despite its advantages, PDMS remains a synthetic and foreign material to cells; consequently, PDMS membranes require protein coatings to enhance their biocompatibility. For these reasons, the following section focuses on PDMS fabrication and surface modification methods.

### PDMS membrane fabrication methods

In addition to its high elasticity, optical transparency, and biocompatibility, PDMS is an elastomer and a standard material due to its high mechanical stability, low chemical reactivity, and low thermal conductivity. However, the composition and intrinsic stiffness of PDMS differs greatly from those of the native ECM^3^, impacting cellular growth and adhesion. Furthermore, PDMS hydrophobicity hinders biomolecule attachment and protein adsorption^79^. Nevertheless, due to the gas permeability of PDMS, it is a suitable biochip material because its conditions resemble aerobic physiological conditions^80^. Gas permeability can also be a drawback since the absorption and adsorption of small molecules may distort the environment^3^. The permeability may also compromise the evaluation of effective drug concentrations or design of anaerobic environments, such as in a gut-on-a-chip for obligate anaerobic bacteria coculture. Several approaches have been proposed to address the issue of drug absorption by PDMS. One approach involves the addition of a PDMS-PEG block copolymer and subsequent pretreatment with the drug at a high concentration prior to performing experiments. This method has been shown to significantly reduce PDMS drug sequestration for four of the five drugs tested^81^. However, the complexity and lack of knowledge on polymeric drug absorption remain a challenge, as it appears to be largely independent of the material’s chemical properties. To better understand and address this challenge, researchers have turned to computational methods, such as modeling, to clarify and minimize drug absorption in microfluidic channels. Shirure and George developed a two-constraint 1D model that considers drug convection, dissolution, and diffusion to minimize drug absorption in a channel^82^. Their work highlights the potential of using computational methods to tackle this complex problem.

PDMS membranes are a versatile and promising element for OOCs, and recent developments in membrane fabrication technology have created new avenues for in vitro research on human physiology and disease. However, traditional PDMS microfluidic device manufacturing techniques, including soft lithography, may involve drawbacks, such as high costs and limited scalability. Therefore, to overcome these limitations, novel fabrication techniques are being developed, including electrospinning, surface modification, additive manufacturing, and templating. Using 3D printing with PDMS, it is possible to produce microfluidic devices with complex geometries and features that are difficult to obtain with traditional fabrication methods. These techniques may enhance the performance of PDMS-based membranes and broaden their applications in the fields of tissue engineering, drug discovery, and personalized medicine^83,84^.

#### Soft lithography

**Fig. 5:**
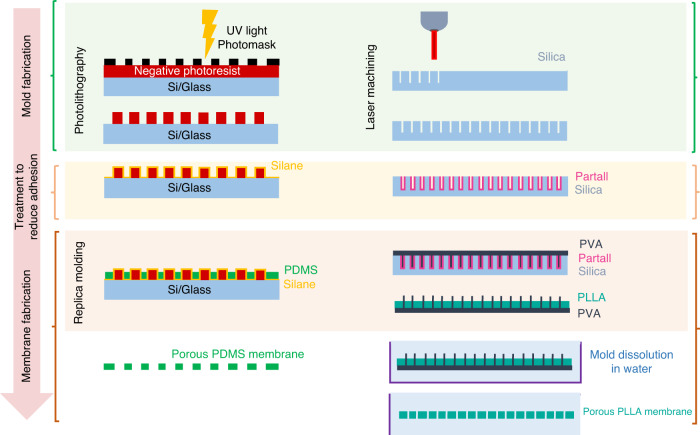
**Porous membrane fabrication processes can be divided into the following main steps: mold fabrication, treatment to reduce adhesion, and membrane fabrication.** Left: a widely implemented technique is photolitographic mold fabrication and posterior replica molding. Right: laser machining mold fabrication to create dissolvable molds is a novel fabrication approach

Submicron features are key for cell culture because they allow cell‒cell communication and physical contact^20^, while thin membranes improve biomolecule transport and nutrient transfer rate^85^. Due to the diffraction limits of conventional photolithography (Fig. 5, left), the production of PDMS membranes with pore sizes and thicknesses smaller than 5 μm is difficult^14^. High-resolution photomasks (<2 μm) for silicon mold fabrication are available but are expensive. Maskless laser direct writing techniques provide an alternative solution to overcome this limitation and provide flexibility concerning micron-scale pattern design and 3D geometries^86,87^. Moreover, as proposed by Le-The and collaborators, photolithography can be combined with methods such as reactive ion etching to pattern submicron photoresist (PR) arrays with photolithography over a sacrificial PR layer^88^. In this method, the arrays are covered with a PDMS:hexane 1:10 solution and spin-coated at 6000 rpm for 3 min. After curing, the PDMS:hexane layer is etched using sulfur hexafluoride and oxygen to open through holes (pores). The membrane is later adhered to a supporting ring, and the sacrificial PR layer is dissolved in acetone. This method achieved membrane thicknesses down to 600 nm due to the high spinning speed^88^. Molds can be alternatively fabricated using the deep reactive ion etching (DRIE) method^89^. Stucki et al. fabricated a 3.5-μm-thick, porous PDMS membrane by micro structuring-lamination, in which the PDMS prepolymer was poured and pressed between a silicon mold with DRIE-structured micropillars and a thin polyethylene sheet^89^. The resulting membrane was implemented in an alveoli-on-a-chip, achieving a total local thickness lower than 10 μm, including the seeded cells^48^.

Thin membranes are more difficult to handle because the risk of tearing increases with pore amount and size. To handle thin membranes, many fabrication protocols involve mold (e.g., SU-8 mold) pretreatment or the use of sacrificial layers to prevent membrane adhesion to the mold, which is usually hydrophilic. Silanization is a treatment that prevents PDMS from sticking to the silicon mold, allowing membranes to easily peel^42,50,90^. Soluble sacrificial layers, such as polyacrylic acid (PAA), have also been used to facilitate porous membrane release, with a transfer success rate of over 85%^14^. Polyvinyl alcohol (PVA) has also been used as a sacrificial layer for PDMS structures^91^. In this regard, Pensabene and collaborators proposed a method to create sacrificial PVA nanoneedles to fabricate a poly (L-lactic acid) (PLLA) membrane for human umbilical vein endothelial cell (HUVEC) culture. The method uses femtosecond laser machining to form a silica wafer with 1-μm-diameter pores, which is then coated with a water/alcohol-based PVA release agent known as Partall® Film #10 (Fig. 5, right). Then, a sacrificial layer of PVA nanoneedles is replica molded (needle final length of 10 μm). First, transparent PLLA is spin-coated on top of the PVA nanoneedle array and left to dry. Then, the sacrificial PVA array is immersed in deionized water to dissolve and help the PLLA membrane release^20^. After de-molding from the reusable silica mold, the PVA sacrificial array displayed over 90% perfect release of nanoneedles. Scanning electron microscopy (SEM) analysis revealed that this method achieved a pore diameter of 1.858 ± 0.33 μm.

#### Pore formation via high-pressure saturated steam

**Fig. 6:**
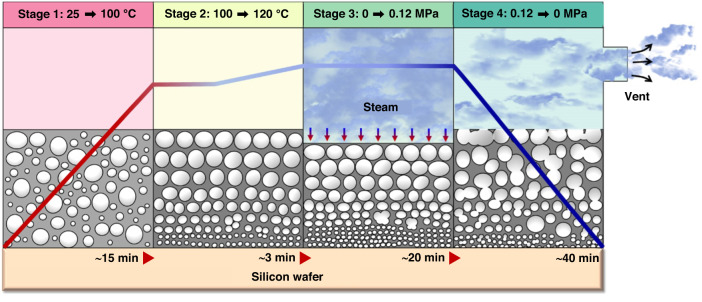
**Porous PDMS membrane fabrication via high-pressure saturated steam method proposed by Jang et al. (2019).** Schematic illustration of 4 stages: 1st—initial heating process to 100 °C, 2nd—temperature increase from 100 °C to 120 °C for pressurization, 3rd—pressurization at 0.12 MPa for 20 min, 4th—pressure release for 40 min. Image from Fig. 1 reference^79^

Pore formation via high-pressure saturated steam has been explored by Jang and collaborators via an autoclaving cycle^79^. This method combines temperature, pressure, and moisture to generate micron-scale bubbles that become pores once PDMS has been cured (Fig. 6). The process reduced the membrane thickness, which enhanced the membrane’s physical properties, increasing Young’s modulus, roughness, and air permeability^79^. Membranes with decreasing thickness possessed a higher number of pores with small pore sizes (<5 μm) and thus were densely packed. Furthermore, the increased surface area notably enhanced platelet, DNA, and collagen adhesion, although the molecules concentrated in the edges of the pores since the increased roughness prevented the molecules from entering the inner pore walls.

#### Alternative methods: 3D printing, electrospinning, and others

**Fig. 7:**
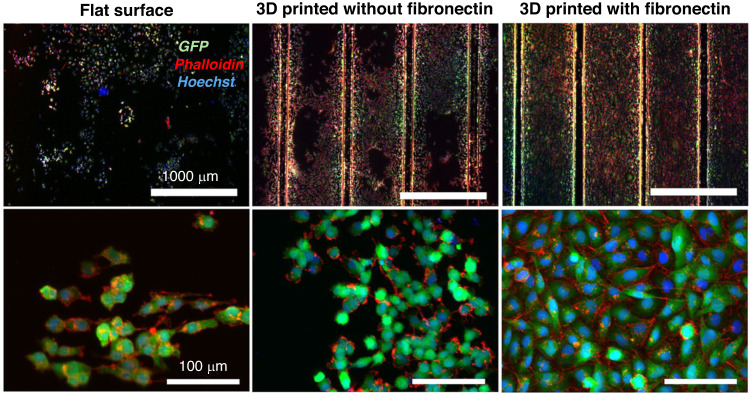
PDMS 3D printing improves cell adhesion and spreading due to uneven surfaces. Immuno-images of adhered cells on cast and 3D-printed surfaces. Image from Fig. 6 reference^62^

Alternative techniques include PDMS 3D printing and the use of porogens (dissolvable particles such as salt and sugar)^92^ and particles (glass microspheres)^93^ for micropatterning. A study conducted by Ozbolat and collaborators found that PDMS 3D printing improved its mechanical properties compared to cast samples (Fig. 7). Five mixtures of low viscosity (SE 1700) and shear thinning (Sylgard 184) PDMS in different ratios were tested in the transverse and longitudinal directions since the printing direction affects the mechanical properties in additive manufacturing. The increase in ultimate strength and failure strain was attributed to the decreased porosity and bubble entrapment during extrusion. PDMS 3D printing was found to improve cell adhesion by ~90% compared to flat cast samples^62^. The uneven surfaces produced in 3D-printed PDMS facilitated cell adhesion and spreading, whereas cells in cast samples formed aggregates and presented a more rounded morphology. 3D printing enables prototyping and may enable the creation of membranes with novel complex geometries^94^.

To easily extend from single-organ OOC to multiorgan OOC (or more specifically, from double-compartment to multicompartment), Lei et al. demonstrated an OOC fabrication method using 3D printing of PDMS prepolymer onto a nanofiber membrane (approximate thickness 30 μm and pore size <5 μm)^95^. Compared to mechanical fixation previously described in the literature, this method achieves better control of material deposition and more stable bonding between the nanofiber membrane and microchannels. Furthermore, a PDMS-based OOC was produced using electrospinning methods. To create movable membrane cavities, pillar cavities, and porous scaffolds important for OOC design, PDMS was employed in a manufacturing approach presented by Qiu et al.^96^ that combines electrospinning and 3D printing. This approach achieved electrospun nanofibers ~25 μm thick with a porosity of 20.42%.

Complicated PDMS structures have been built through sacrificial templates. To fabricate a PDMS membrane that achieves cell adhesion and viability assessment, Keshtiban et al. created a time- and cost-efficient porous membrane in which a sacrificial layer (PVA) and an O_2_ plasma surface treatment were employed^97^. Ferreira et al. presented a novel way to construct intricate multilayered PDMS fluidic devices with integrated microactuators suited for OOC applications^98^. In their study, cells were seeded on a PET membrane (thickness 16 μm, pore size 8 μm), and featureless PDMS foil sheets were used for actuation. The authors noted that silanization of the PET membrane with bis-amino silane did not affect cellular homeostasis or induce cytotoxicity.

Due to its advantages, PDMS has become an appealing material for the fabrication of various microfluidic chips. However, because of its hydrophobic nature, PDMS is not an adequate substrate for cell attachment and growth. The following section briefly discusses the mechanisms involved in cell-membrane adhesion as well as the materials often used to enhance cell growth on PDMS membranes.

### Protein and hydrogel membrane coatings

This section addresses the main biomaterials used to enhance synthetic membrane biocompatibility or promote cell interactions with the substrate. Emphasis is placed on commonly implemented and well-characterized biomaterials, such as collagen, fibronectin, and hydrogels. Additionally, biomaterial patterning is introduced as a means to delimit the desired areas for cell growth in membranes or substrates.

#### Proteins for OOC membrane coating

Collagen and fibronectin are two proteins mainly implemented individually to mimic the ECM in vitro. Collagen, the most abundant protein in the native ECM^99^, is a stiff, tension-bearing fiber that functions as a stabilizer^100^. It acts as an endothelial cell propagation agent to increase cell yield, survival, and proliferation^101^. Consequently, collagen has been used in various organ models, such as alveoli^48^, spinal cord^102^, and intestine^103,104^. Fibronectin is the second most abundant protein in the native ECM and a major tissue component that functions as a template during early development as well as wound healing^100,105^. Fibronectin is also a mechano-regulator protein that, when unfolded, presents arginine-glycine-aspartic acid (RGD) integrin-binding sites that bind to the cytoskeleton, a cellular structure that enables cell contraction^106^. This “master organizer” protein functions as a primary structure that later promotes the deposition of native collagen I and elastin, and therefore, it is used to form soft connective and elastic tissues, such as skin, lungs, ligaments, and tendons^107^. Fibronectin has been used to coat synthetic membranes in a wide variety of OOCs, such as lung^4,24,42^, intestine^108,109^, blood vessels^44^, kidney^110^, and placenta^40^. Despite having specific roles, collagen, and fibronectin have been used for the same kinds of cells and appear to have functionality overlaps. The choice of one or another may also involve pricing, as well as the practical considerations in wet laboratories (solution preparation, spreading, and consistency).

The combination of collagen and fibronectin results in synergistic structural support that has been shown to promote cell adhesion and proliferation^111^. Fibronectin initially directs the hierarchical assembly of the ECM and regulates the localization of collagen, and later, collagen stabilizes the structure^100^. Cell contractility and migration are enhanced through fibronectin-collagen interactions^112,113^ due to durotaxis or cell migration up stiffness gradients^114^. Collagen and fibronectin combinations have been implemented for epithelial or vascular cell lines in alveoli^24^, kidney^110,115^, and intestine^108^. Matrigel ®, an ECM-based, murine-derived hydrogel, is a widely used matrix since it resembles the epithelial basement membrane. Matrigel ® has been combined with collagen I to culture epithelial lung cells^90^ and is common for applications related to the gastrointestinal tract, such as the gut^34,41,116,117^, intestine^33,118^ and colon^119^, with cell lines such as Caco-2 and human intestinal microvascular endothelial cells (HIMECs). Matrigel® also contains proteins such as laminin and heparan sulfate proteoglycan, both ECM structural elements that influence cellular phenotypes and FSS sensing, respectively^120,121^. However, its variable growth factor composition can undesirably affect gene expression; thus, natural proteins are more reliable coatings^122,123^.

**Fig. 8:**
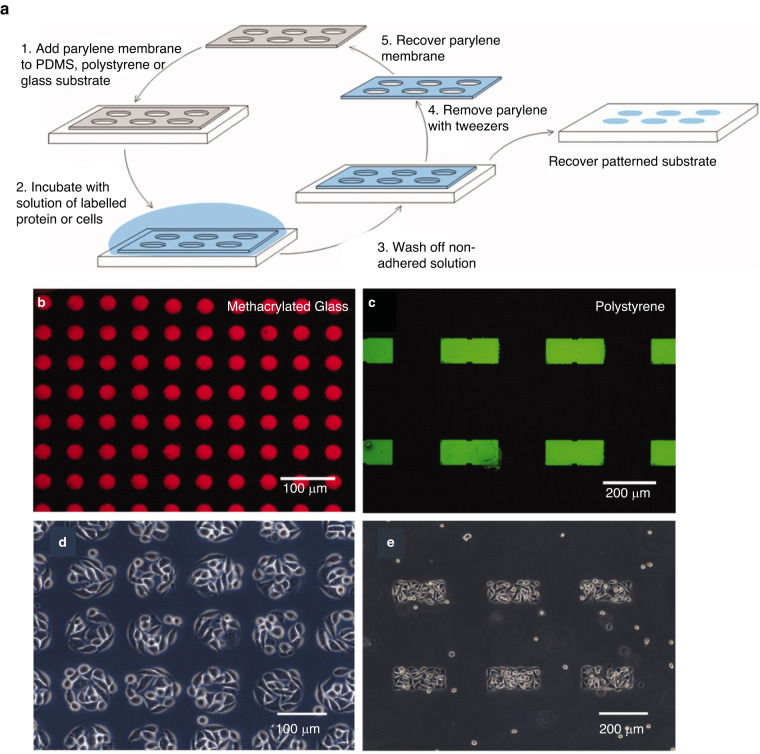
**Protein and cell patterning.** **a** Schematic of the patterning process with reusable parylene stencil. **b**, **c** Fluorescent protein pattern. **d**, **e** Cell patterning (NIH-3T3 fibroblasts) on PDMS, substrate initially coated with FN to increase adhesion^126^. Image adapted from Figs. 2, 4, and 6 from reference^126^

Proteins can be coated on membranes as a single layer or locally in specific shapes or patterns. The selective transfer is achieved via microcontact printing (μCP) or microfluidics and provides geometrical cues to which cells (e.g., cardiac tissue or muscular fibers) respond through changes in direction and alignment^124^. μCP protein patterns can reach sizes as low as 0.5 μm; however, pattern integrity depends on the feature size and the chemical nature of the membrane, as larger features and hydrophobic surfaces present more robust binding^125,126^. A study conducted by Wright and collaborators achieved fibronectin patterning onto PDMS and polystyrene with reusable 10-μm-thick parylene-C stencils (Fig. 8)^126^.

#### Hydrogels for OOC membrane coating

Hydrogels effectively mimic the ECM’s chemical composition and mechanical properties^127^ and can therefore biophysically stimulate cell differentiation and alignment^128^. Hydrogels are 3D polymeric networks that swell in aqueous solutions and remain insoluble due to their crosslinks^129^ and have been widely used in tissue culture to study cell-matrix and cell‒cell interactions^130^. Hydrogel physical and biochemical properties can be tuned by adjusting its composition, degree of polymerization and crosslinking density^131^. Natural hydrogels, such as alginate, chitosan, and hyaluronic acid (HA) are biocompatible; e.g., HA possesses the receptor CD44, which results in cell adhesion when bound^74^. Semisynthetic hydrogels, such as gelatin methacryloyl (GelMA), contain natural and synthetic components. Gelatin is a product of collagen hydrolysis that contains cell-attaching sequences, such as RGD and matrix metalloproteinase peptide motifs, through which cells can attach, proliferate and spread. Methacrylamide and methacrylate are synthetic elements that improve thermostability and photocrosslinking^131^.

Given their intrinsic brittleness, hydrogels are mostly fabricated on a supporting substrate (e.g., synthetic membranes or films, glass); nevertheless, they can be functionalized or combined with other materials, such as copolymers, to create free-standing membranes^132^. Hydrogels can be microfabricated by photopatterning and micropatterning, stereolithography, micromolding, microfluidics-enabled viscous fingering, and bioprinting^130^. Stimuli-responsive hydrogels can be actuated or patterned via pH, temperature, ionic strength, and electric or magnetic fields^129^. Poly(N-isopropylacrylamide) (PIPAAm) is a thermoresponsive hydrogel that switches its solubility and aggregation in an on-off fashion due to its lower critical solution temperature (LCST). PIPAAm displays hydrophobic behavior at 37 °C (shrinkage and ligand exposure for cell binding), while below its LCST (32.1 °C), its surface becomes hydrophilic and swells^133^. Thermally responsive membranes can be used for potential on-chip applications, such as cell monolayer release after the temperature is decreased from 37 to 20 °C^133^. Another possibility is filtration since the membrane can be reversibly switched to block or allow molecule diffusion^127^. Another stimulus-responsive hydrogel is chitosan, the polymerization of which can be altered through protonation. A pH-responsive chitosan membrane is further discussed in the next section. Although hydrogel properties (e.g., tensile strength and water content) can be tuned, there may be a tradeoff concerning some of their key advantages. An increase of over 15% in GelMA’s methacryloyl (synthetic component) content decreases its degradability and negatively impacts cellular growth^130^. Another example is a gelatin-hydroxyphenylpropionic acid (Gtn-HPA) conjugate, as the reaction catalysts hydrogen peroxide (H_2_O_2_) and horseradish peroxidase (HRP) increase the hydrogel’s stiffness, which negatively impacts cell attachment and proliferation^56^.

We have discussed the reasoning and advantages of biological coatings on synthetic membranes. Another alternative is fabricating membranes created solely from biological materials, such as protein ECM components; this provides an alternative method to create more physiologically representative environments. Protein-based membranes, their fabrication methods, and incorporation into OOCs are discussed in the following section.

## Biological ECM protein membranes

The extracellular matrix can be structurally divided into two parts. One is the compact and porous basement membrane, composed of proteins such as collagen IV, fibronectin, and laminin. The other part is the dense and hydrophilic interstitial matrix, which is composed of proteoglycans and fibrillar (type I) collagens^134^. The ECM is not only relevant as a support structure but is also crucial for basic cell adhesion, growth^19^, and common cellular functions, such as signaling, differentiation, and phenotype definition^6^. Cells respond to the surrounding ECM by secreting proteins, as well as adapting to and remodeling their environment to comply with their functions^135^. Thus, the ECM plays a bioactive role in homeostasis and influences the cellular response in health and disease^3^.

ECM membranes are those constituted by densely packed protein fibers present in the native extracellular matrix. These membranes provide a large surface contact area for cells^136,137^, as well as mechanical and chemical cues similar to those found in physiological environments. Because of their native-protein composition, ECM membranes are more biologically representative, as they can generate better cellular responses than those of synthetic polymeric membranes. Membrane composition can be modified through protein content to adjust physical aspects (e.g., stiffness, permeability, transparency, and porosity) or chemical characteristics to which cells respond (e.g., adhesion and organization) to provide cells with instructive cues to modify their phenotype, as well as regulate or dysregulate their behavior^19^. Integrins are transmembrane proteins that transduce external signals and experience conformational changes. Integrins interact with ECM proteins (collagen, fibronectin, laminin) and affect cell shape through their connections with the cytoskeleton^138^. The advantages of ECM membranes in cell culture were demonstrated by Wang et al., who compared colonic cell culture on three different fibronectin-coated devices. A membrane-less device showed a decline in cell viability after Day 1 and maintained round cell morphology, which indicates a lack of differentiation^139^. The viability further decreased to 76% on Day 5, indicating an early apoptotic phase^136^. A device with a Transwell ® polyester membrane (10 μm thickness, 0.4 µm pore size) presented slightly improved viability (85% on Day 5), and the cell morphology changed from round to elliptical after confluency, which indicates partial cell spreading. The small difference in viability could be attributed to the more dynamic environment introduced by the membrane. However, the cellular death phase was soon observed. The third device comprised a 15 μm thick collagen membrane (with pore diameter ~10 μm), and in contrast with the other two devices, presented no decrease in viability up to Day 5. Moreover, the dynamic environment and native-like material induced several types of cell morphologies, including round, squamous, and intertwined with the collagen fibers, which is an indicator of close cell-membrane interaction^136^. The dense reticular structure of this membrane better supported growth and viability as the cells further differentiated in the presence of a more biological environment. The viability on Day 5 showed a 10% improvement over that of the polyester membrane. Furthermore, the cells cultured on the collagen membrane displayed several-fold higher tight junction proteins (F-actin, ZO-1, and ezrin) than that of the other two devices. Actin is relevant for cell division, while the tight junction protein ZO-1 regulates barrier function, and ezrin aids in cell adhesion and migration. A major challenge with this ECM membrane is its fabrication process, as it is difficult to pour and spread the ECM solution onto the mold due to the viscosity and surface tension^136^. Additionally, the proposed method involves mechanical peeling, which is technically challenging for thin membranes due to the risk of tearing. As an alternative, Mondrinos and collaborators proposed ECM membrane fabrication using vitrification through cycles of drying and hydration of ECM hydrogels. Through this method, a collagen membrane with a thickness of 20 µm and nanoscopic pores ( ~ 250 nm) was obtained^19^. Despite the dense network of randomly oriented fibers, the membrane was optically clear, and it presented a lower light absorbance than that of polyester Transwell ® membranes. The composition was then modified by adding Matrigel® to collagen to enhance the transparency of the membrane; however, this also resulted in a reduction in Young’s modulus due to the decrease in fibrous collagen content. Both membranes presented low gas permeability because of their densely packed fibrous structure. To increase the membrane’s pore size (700 nm) and consequently, the permeability, alginate was added as a sacrificial material, which after membrane gelation was removed using DDI water. Collagen membranes exhibit several advantages, such as long-term stability, maintenance of structural integrity, and resistance to proteolytic degradation^19,140^. Nevertheless, when the membrane is hydrated, its resistance to bending and stiffness are altered, and it becomes a more compliant and softer gelatinous matrix. A similar mechanical behavior (collagen membrane Young’s modulus ~660 kPa) is observed in tissues, such as the lens capsule (0.3–2.4 MPa); this behavior is advantageous in applications that require actuation or membrane deformation.

ECM membrane-on-a-chip (in situ) fabrication is an innovative approach that prevents membranes from being exposed to ambient conditions and simplifies device fabrication^141,142^. Herland and collaborators used this on-chip fabrication approach for a blood‒brain–barrier (BBB) model, where a cylindrical lumen membrane was fabricated through viscous fingering displacing a viscous liquid (collagen I solution) with a less viscous liquid (culture medium)^143^. The model employed microfluidics to deliver cells with TNF-α to study the inflammatory response via the cytokine release profile. A square-shaped microchannel was filled with collagen I, and then hydrostatic medium flow was applied to the finger through the solution, creating a cylindrical lumen after gelation. The model cocultured three cell types; astrocytes were mixed with collagen prior to viscous fingering, and the lumen was later lined with pericytes (seeded statically) and endothelial cells (seeded under fluid flow) to create an endothelial layer. As a result of multilayer cell seeding, ECM-embedded astrocytes extended processes toward the endothelium, pericytes tightly associated with the basement membrane, and the endothelial cells lining the collagen cylinder secreted their own basement membrane and formed a microvessel-on-chip^143^. The luminal 3D conformation presented a homogeneous collagen network that mimicked the subendothelial brain space more accurately than planar models. Moreover, the membrane presented an improved barrier function compared to that of monolayers and cocultures on Transwell ® inserts.

It is difficult to replicate physiological interactions between the vascular endothelium and parenchymal sides (native interface) with organ-on-chip technology, mostly due to the restrictions of the microfluidic chip architecture^144^. Porous membranes (PC-, PDMS-, or PET-based) are frequently employed to mimic the functionality of the basal lamina to address this problem. These membranes are stable but present issues when simultaneously mimicking physiological interactions and signaling. Ideally, in an OOC, the barrier between the parenchymal channel and the endothelial channel should allow direct cell‒cell interaction without the interference of a nonphysiological membrane^70^. By using a temporary membrane, it is possible to create a coculture of different cell types on the chip without using an artificial or synthetic membrane. Endothelial cells develop natural basal lamina when the artificial membrane separating two cell types is removed, resulting in a stable basement membrane between parenchymal and endothelial cells^143^.

**Fig. 9:**
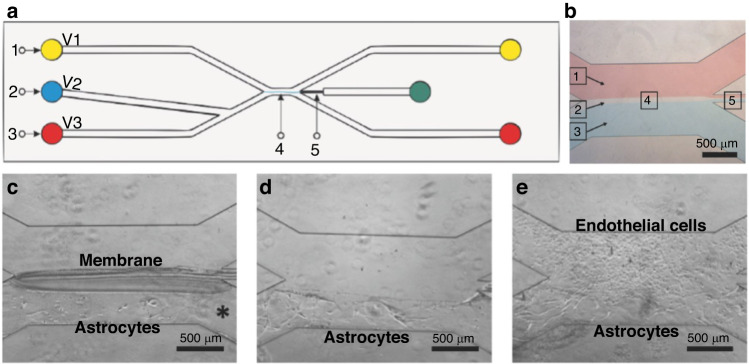
**Tibbe et al. (2018) in situ chitosan membrane fabrication.** **a** Chip design where V1 is the inlet for a basic solution (pH 9.6), V2 the inlet for the chitosan solution, and V3 the inlet for the neutral (pH 7) solution. The chitosan membrane forms at 4, whereas 5 is the outlet for the chitosan solution. **b** Bright field image of the basic solution (red), chitosan solution (transparent), and neutral solution (blue). The experiment is depicted in **c**–**e**. After membrane polymerization, astrocytes are seeded in the bottom chamber (**c**), the chitosan membrane is later removed (**d**) and last, endothelial cells are seeded in direct contact with astrocytes for coculture (**e**). Image adapted from Figs. 1 and 3 from reference^70^

Another BBB-on-a-chip that implemented in situ fabrication achieved a temporary chitosan membrane using microfluidics. The membrane functions as a support for astrocytes; once astrocyte culture is established, the membrane is removed to coculture endothelial cells in direct contact with astrocytes (Fig. 9e). Chitosan is a pH-responsive, chitin-derived polysaccharide^145^ that resembles the ECM stiffness, water content, and cell adhesion. Membrane fabrication was carried out leveraging chitosan’s pH-responsiveness via interfacial polymerization. Carbonate (neutral, pH 7.0) and phosphate (basic, pH 9.6) buffers were co-flowed on a microfluidic device (Fig. 9b). The chitosan membrane is formed between the interface between the buffers. Chitosan is injected and contacts the carbonate, which protonates chitosan and makes it soluble. Then, chitosan polymerizes through deprotonation when it contacts the basic phosphate buffer. After seeding astrocytes on one side of the membrane, chitosan was removed via protonation through an acetic acid solution (pH 5.0) (Fig. 9c, d). Later, endothelial cells were seeded in direct contact with astrocytes, achieving membrane-free BBB coculture^70^. The method achieves close cell association, induces endothelial cells to produce a native and stable basement membrane, and is not cytotoxic. As a natural hydrogel, chitosan provides excellent support, but its biocompatibility and mechanical properties can be improved when combined with proteins such as collagen^146^. ECM and ECM-like biomaterial membrane fabrication can greatly improve cell viability on-chip and generate more physiological responses than those of synthetic membranes. The implementation of responsive materials, such as chitosan, also provides an alternative method to create temporary barriers for coculturing different cell types that contact each other without intermediaries.

## Summary and discussion

OOCs remain a powerful in vitro platform to study biological responses, impact disease treatments, and replace animal testing for drug discovery. The demand for more physiological environments to obtain representative results increases when body-on-a-chip platforms are favored for more complex applications, such as personalized medicine. This emphasizes the relevance of physiological models to study defective cell signaling that leads to pathology and the efficacy of drug candidates. In this context, membranes significantly increase OOC representativity to effectively mimic in vivo responses; they enable cell communication through pores or act as biological supports to enable cell-matrix interactions. Depending on the desired cell type and application, the membrane characteristics (porosity and thickness), material (biocompatibility, elasticity, transparency), fabrication method (feasibility, and reproducibility), and topography (micropillars or grooves) must be carefully selected to achieve direct interaction between the cells and membrane. In this section, we outline and summarize the importance of various membrane parameters that were previously described with examples.

The biophysical forces in the microenvironment and the membrane material properties influence cell growth and development. Continuous perfusion and FSS enable inhibitory factor removal and 3D morphogenesis, while cyclic mechanical stimulation influences cell contractility and alignment. The combination of both stimuli leads to a more accurate replication of organ function. On the other hand, membrane thickness directly influences cell communication through the pores. Porosity in turn regulates the interaction between chambers and influences cell adhesion and then differentiation. Relief features influence cell alignment, attachment, and ECM deposition. The chip design and fluidic components are also fundamental for membrane shape and tuning. Stacked chambers are often used with PDMS porous membranes for cell communication, while lateral chambers often imply mechanical stimulation. Regardless, pore size and thickness are adjusted as a function of the cell type and application.

The introduction of Transwell ® inserts established the separation of the apical and basolateral contents of a well by means of a membrane (PET, PC). Today, Transwells ® are the gold standard for epithelial transport due to their robustness and reproducibility^147^ and are considered a baseline for comparison with microfluidic devices with similar basal‒apical conformations^27,116,136,148^. Transwell® cell culture, however, allows cell polarization of a monolayer^149^ and lacks liquid flow, both of which are nonphysiological conditions that do not support tissue-specific differentiation^147,150^. To address the absence of liquid flow, hybrid insertable devices^34^ allow longer-term cultures, emphasizing the relevance of fluidic flow for biological systems. Ultimately, the demand for even more dynamic environments (e.g., simultaneous liquid flow and mechanical stimulation) resulted in the development of OOCs that achieved longer-lasting viability and superior physiological mimicry than their static equivalent (Transwells ®). OOCs also introduced a wide diversity of designs, membranes, cell types, and materials, which benefited the compatibility with external equipment (e.g., pumps and probes for sensing) and device element customization, e.g., incorporation of electrodes for real-time monitoring and stimulation^63,151^. On the other hand, these diversities pose challenges concerning standardization and reproducibility.

Alternatively, fabricating membranes poses several challenges involving robustness, comparison, and standardization. Membrane fabrication entails characterization and testing prior to incorporation into a device. The lack of a gold standard particularly affects those protein/ECM-based membranes, as synthetic membranes can be compared to Transwell ® for static studies. This renders many studies comparing their membrane material with itself in different conformations, thicknesses, or porosities only or contrasting with a static well culture, which despite being a standard becomes more unsuitable as OOCs become more specialized and combine features that vary according to each study’s specific aims.

Commercially available synthetic membranes (PET, PC) provide repeatability and can be readily compared with Transwell® cultures. Therefore, these membranes are implemented in studies of biological functions, such as elucidating specific mechanisms of gene expression^108^ and culturing sensitive primary cell lines. However, their lack of transparency and flexibility hinders microscopy monitoring and mechanical stimulation of the cultured cells, respectively.

Transparent and flexible PDMS is a widely used alternative with well-characterized mechanical and chemical properties^152–154^. PDMS membrane elasticity enables the direct transmission of mechanical stimulation to the cells in OOC. Membrane fabrication methods are greatly focused on photolithography and replica molding (soft lithography), although innovative methods have been proposed. For instance, the application of high-pressure saturated steam using a conventional autoclave enabled the development of synthetic polymeric membranes with pore features smaller than 5 μm. Smaller sizes could be achieved by combining, for example, laser micromachining and sacrificial molds (for example, when a PVA nanoneedle mold was used for porous PLLA membrane fabrication). PDMS chips and membranes are also commercially available. As an example, the S-1 Stretchable Chip marketed by Emulate Inc. provides a PDMS membrane for cell mechanical stimulation. The chip can emulate several organs (liver, kidney, blood vessel) through the culture module, which controls both media flow and stretch, achieving a high transcriptomic similarity of the OOC with its in vivo equivalents. For instance, the S-1 Chip has been reported to accurately emulate the mucus layer physiology of human primary colonic intestinal epithelial cells^119^.

Smart materials enable alternative cell stimulation approaches. For example, thermoresponsive PIPAAm was used in regenerative medicine for cell monolayer release when the temperature was decreased below the LCST. Conductive materials or coatings are also a promising approach for organs that need electrical stimulation, such as cardiomyocytes^155^ or neurons^156^. However, synthetic hydrophobic membranes, including PDMS, require protein coatings or surface treatments to enhance cell adhesion^136^. The protein coating does not compensate for the biochemical composition and mechanical properties (stiffness, topography) of the native ECM, as the bulk material (PDMS) remains unnatural^19^. On the other hand, the composition of ECM membranes created from collagen or chitosan closely mimics the native ECM. Nevertheless, to achieve an accurate physiological response in vitro, membranes should recreate the mechanical stimuli faced by cells in vivo (namely, stiffness and elasticity, which influence their IPR, durotaxis, and adhesion), including suitability for mechanical actuation. The ability to tune the stiffness of the membrane by adjusting its composition or production parameters is also important, as it allows for the creation of membranes with a range of stiffnesses that can mimic the mechanical properties of different types of tissue barriers. Additionally, the membrane should be thin and flexible enough to allow for mechanical stimulation of the cells grown on the membrane through flexing or stretching. Examples of such membranes include PDMS membranes and collagen-elastin (CE) membranes. CE membranes, in particular, have composition and mechanical properties similar to those found in vivo. Their stiffness can be tuned by adjusting the CE ratio, the production mode, and/or parameters such as temperature, allowing for the creation of membranes with stiffnesses ranging from several hundred kPa down to 1 kPa^157^. The mechanical properties of these membranes can be characterized using techniques such as the bulge test and atomic force microscopy (AFM).

Studies involving ECM membranes are predominantly oriented toward analyzing the membrane’s mechanical characteristics, physical properties (e.g., permeability), and cell-membrane interaction to compare the membrane’s physiological representativity with that of standard tissue culture platforms (e.g., petri dish, Transwell ®)^19,149^. Perhaps a greater challenge is the standardization of film-shaped ECM membrane fabrication, which encompasses straightforward methods (pour and dry) that lack reproducibility in parameters such as thickness and protein concentration^19,136^. Due to differential protein behavior in varying environments (e.g., brittle vs. gelatinous consistency in dry and wet environments, respectively), in situ luminal-membrane fabrication is attractive for applications such as the vessel or kidney that benefit from cylindrical membrane conformations that emulate in vivo vascular structures. In this regard, collagen is currently a preferred material over fibronectin due to its wider availability, lower cost, and superiority in promoting proliferation and providing structural support for cells^101^. Fibronectin, on the other hand, promotes the deposition of more flexible proteins, such as elastin and laminin, which are necessary for tissues such as the skin and lungs. ECM membranes, however, are comparable to Transwell ® (synthetic membranes) only when the same experiment is performed and the synthetic membrane is used as a control, as currently there is no readily available ECM membrane standard. Nevertheless, ECM membranes compete with well-established PDMS membranes (and their more standardized techniques), and thus, the development of more robust ECM membrane fabrication methods might level off PDMS dominance in microfluidic devices.

When evaluating the use of porous membranes for cell seeding, it is important to consider the potential use of hydrogels and ECM proteins as alternative surfaces. For example, hydrogels and ECM proteins are particularly useful for creating tubular or cylindrical conformations, such as vessels^143^, in which case lining a channel with the matrix can provide sufficient support for cell growth. However, not all OOC designs rely solely on hydrogels and ECM proteins. Epithelial layers, on the other hand, require a larger surface area for growth and junction formation. In these cases, porous membranes may still be necessary to provide the necessary surface area and support for cell growth.

For 3D vessel self-assembly networks, incorporating a membrane into the chip may not be necessary^158^. The self-assembly of vessel networks can be stimulated in vitro within 3D scaffolds through the coculture of endothelial cells (ECs), vascular mural cells, and cells specific to the tissue of interest. Vascular mural cells, which include smooth muscle cells or pericytes, provide physical support to ECs and generate extracellular proteins such as collagen, laminin, and fibronectin. They also release proangiogenic growth factors (GFs), such as VEGF, FGF, TGF, and angiopoietin, which induce vascularization. For further discussion on the advantages of OOCs with no membranes (e.g., the maximization of cellular interaction), the reader may refer to the articles by Argentiere et al.^159^ and Rahmani Dabbagh et al.^160^.

While coculture can increase the physiological relevance of an OOC device, it can also pose challenges in maintaining cell viability and desired cell type ratios. Coculture with bacteria can be particularly challenging due to bacterial colonization, which can impede cell growth. Therefore, ultimately, the decision to include or exclude synthetic membranes in an OOC design should be based on a careful evaluation of the specific research goals and requirements. If the functions of synthetic membranes, such as mechanical stimulation or cell support/communication, can be achieved by other means (such as using protein or ECM materials), then it may be beneficial to exclude synthetic membranes.

## Conclusions

Membranes in OOCs help provide environments that closely mimic those experienced by cells in vivo. These environments involve mechanical cues, especially shear stress and mechanical stretching, or inherent material characteristics such as stiffness and topography. This review focused on the key membrane characteristics, roles, and materials for different OOCs. PDMS is a preferred material for stretching applications and gene expression studies but often requires protein coatings, whereas ECM membranes that contain proteins can support cell growth for long periods of time and induce more physiological responses but lack robustness and a baseline for comparison. Optimum membranes should be composed of native proteins that are thin enough to enable cell communication and/or contact without trading off the flexibility necessary for mechanical stimulation.

## References

[1] Yang X (2018). Nanofiber membrane supported lung-on-a-chip microdevice for anti-cancer drug testing. Lab Chip.

[2] Bein A (2018). Microfluidic organ-on-a-chip models of human intestine. Cell. Mol. Gastroenterol. Hepatol..

[3] Zamprogno P (2021). Second-generation lung-on-a-chip with an array of stretchable alveoli made with a biological membrane. Commun. Biol..

[4] Felder M (2019). Impaired wound healing of alveolar lung epithelial cells in a breathing lung-on-a-chip. Front. Bioeng. Biotechnol..

[5] Novak R (2020). Robotic fluidic coupling and interrogation of multiple vascularized organ chips. Nat. Biomed. Eng..

[6] Trietsch SJ (2017). Membrane-free culture and real-time barrier integrity assessment of perfused intestinal epithelium tubes. Nat. Commun..

[7] Wang Y, Wang L, Guo Y, Zhu Y, Qin J (2018). Engineering stem cell-derived 3D brain organoids in a perfusable organ-on-a-chip system. RSC Adv..

[8] LIU J-S (2017). Design and validation of a microfluidic chip with micropillar arrays for three-dimensional cell culture. Chin. J. Anal. Chem..

[9] Yazdi MK (2020). Hydrogel membranes: a review. Mater. Sci. Eng. C..

[10] Powers MJ (2002). A microfabricated array bioreactor for perfused 3D liver culture. Biotechnol. Bioeng..

[11] Zheng W, Zhang W, Jiang X (2013). Precise control of cell adhesion by combination of surface chemistry and soft lithography. Adv. Healthc. Mater..

[12] Zorlutuna P, Rong Z, Vadgama P, Hasirci V (2009). Influence of nanopatterns on endothelial cell adhesion: enhanced cell retention under shear stress. Acta Biomater..

[13] Su N (2017). Fibrous scaffolds potentiate the paracrine function of mesenchymal stem cells: a new dimension in cell-material interaction. Biomaterials.

[14] Quirós-Solano WF (2018). Microfabricated tuneable and transferable porous PDMS membranes for organs-on-chips. Sci. Rep..

[15] Borenstein JT (2002). Microfabrication technology for vascularized tissue engineering. Biomed. Microdevices.

[16] Renth AN, Detamore MS (2012). Leveraging ‘raw materials’ as building blocks and bioactive signals in regenerative medicine. Tissue Eng. Part B. Rev..

[17] Neiman JAS (2015). Photopatterning of hydrogel scaffolds coupled to filter materials using stereolithography for perfused 3D culture of hepatocytes. Biotechnol. Bioeng..

[18] Zhang Y (2009). Tissue-specific extracellular matrix coatings for the promotion of cell proliferation and maintenance of cell phenotype. Biomaterials.

[19] Mondrinos MJ, Yi Y-S, Wu N-K, Ding X, Huh D (2017). Native extracellular matrix-derived semipermeable, optically transparent, and inexpensive membrane inserts for microfluidic cell culture. Lab Chip.

[20] Pensabene V (2016). Ultrathin polymer membranes with patterned, micrometric pores for organs-on-chips. ACS Appl. Mater. Interfaces.

[21] Haycock, J. W. 3D cell culture: methods and protocols. *In*: Methods in Molecular Biology. 695 (2011). 10.1007/978-1-60761-984-0_1 21042962

[22] Rahimnejad M (2022). Engineered biomimetic membranes for organ-on-a-chip. ACS Biomater. Sci. Eng..

[23] Lawrence BJ, Madihally SV (2008). Cell colonization in degradable 3D porous matrices. Cell Adh. Migr..

[24] Jain A (2018). Primary human lung alveolus-on-a-chip model of intravascular thrombosis for assessment of therapeutics. Clin. Pharmacol. Ther..

[25] Sung JH (2013). Microfabricated mammalian organ systems and their integration into models of whole animals and humans. Lab Chip.

[26] Marin TM (2019). Acetaminophen absorption and metabolism in an intestine/liver microphysiological system. Chem. Biol. Interact..

[27] Kulthong K (2020). Microfluidic chip for culturing intestinal epithelial cell layers: characterization and comparison of drug transport between dynamic and static models. Toxicol. In Vitro.

[28] Chung HH, Mireles M, Kwarta BJ, Gaborski TR (2018). Use of porous membranes in tissue barrier and co-culture models. Lab Chip.

[29] Cucullo L, Hossain M, Puvenna V, Marchi N, Janigro D (2011). The role of shear stress in Blood-Brain Barrier endothelial physiology. BMC Neurosci..

[30] Jang K-J (2011). Fluid-shear-stress-induced translocation of aquaporin-2 and reorganization of actin cytoskeleton in renal tubular epithelial cells. Integr. Biol..

[31] Kaarj K, Yoon J-Y (2019). Methods of delivering mechanical stimuli to organ-on-a-chip. Micromachines.

[32] Delon LC (2019). A systematic investigation of the effect of the fluid shear stress on Caco-2 cells towards the optimization of epithelial organ-on-chip models. Biomaterials.

[33] Jalili-Firoozinezhad S (2019). A complex human gut microbiome cultured in an anaerobic intestine-on-a-chip. Nat. Biomed. Eng..

[34] Shin W, Hinojosa CD, Ingber DE, Kim HJ (2019). Human intestinal morphogenesis controlled by transepithelial morphogen gradient and flow-dependent physical cues in a microengineered gut-on-a-chip. iScience.

[35] Marrero D (2021). Gut-on-a-chip: mimicking and monitoring the human intestine. Biosens. Bioelectron..

[36] Leung CM (2022). A guide to the organ-on-a-chip. Nat. Rev. Methods Prim..

[37] Lembong J, Lerman MJ, Kingsbury TJ, Civin CI, Fisher JP (2018). A fluidic culture platform for spatially patterned cell growth, differentiation, and cocultures. Tissue Eng. Part A.

[38] Trieu, D., Waddell, T. K. & McGuigan, A. P. A microfluidic device to apply shear stresses to polarizing ciliated airway epithelium using air flow. *Biomicrofluidics* **8**, 064104 (2014). 10.1063/1.4901930 PMC4235625 25553181

[39] Faley SL (2019). iPSC-derived brain endothelium exhibits stable, long-term barrier function in perfused hydrogel scaffolds. Stem Cell Rep..

[40] Blundell C (2018). Placental drug transport-on-a-chip: a microengineered in vitro model of transporter-mediated drug efflux in the human placental barrier. Adv. Healthc. Mater..

[41] Kim HJ, Huh D, Hamilton G, Ingber DE (2012). Human gut-on-a-chip inhabited by microbial flora that experiences intestinal peristalsis-like motions and flow. Lab Chip.

[42] Huh D (2012). A human disease model of drug toxicity-induced pulmonary edema in a lung-on-a-chip microdevice. Sci. Transl. Med..

[43] Musah, S., et al. Mature induced-pluripotent-stem-cell-derived human podocytes reconstitute kidney glomerular-capillary-wall function on a chip. *Nat. Biomed. Eng*. **1**, 0069 (2017). 10.1038/s41551-017-0069 PMC5639718 29038743

[44] van Engeland NCA (2018). A biomimetic microfluidic model to study signalling between endothelial and vascular smooth muscle cells under hemodynamic conditions. Lab Chip.

[45] Ishikawa T, Sato T, Mohit G, Imai Y, Yamaguchi T (2011). Transport phenomena of microbial flora in the small intestine with peristalsis. J. Theor. Biol..

[46] Shin W, Kim HJ (2018). Intestinal barrier dysfunction orchestrates the onset of inflammatory host-microbiome cross-talk in a human gut inflammation-on-a-chip. Proc. Natl Acad. Sci. USA.

[47] Marsano A (2016). Beating heart on a chip: a novel microfluidic platform to generate functional 3D cardiac microtissues. Lab Chip.

[48] Stucki JD (2018). Medium throughput breathing human primary cell alveolus-on-chip model. Sci. Rep..

[49] Chen K (2018). Role of boundary conditions in determining cell alignment in response to stretch. Proc. Natl Acad. Sci..

[50] Schneider O, Zeifang L, Fuchs S, Sailer C, Loskill P (2019). User-friendly and parallelized generation of human induced pluripotent stem cell-derived microtissues in a centrifugal heart-on-a-chip. Tissue Eng. Part A.

[51] Varone A (2021). A novel organ-chip system emulates three-dimensional architecture of the human epithelia and the mechanical forces acting on it. Biomaterials.

[52] Ballermann BJ, Dardik A, Eng E, Liu A (1998). Shear stress and the endothelium. Kidney Int. Suppl..

[53] Sinha R (2016). J. Endothelial cell alignment as a result of anisotropic strain and flow induced shear stress combinations. Sci. Rep..

[54] Ribas J (2016). Cardiovascular organ-on-a-chip platforms for drug discovery and development. Appl. Vitr. Toxicol..

[55] Varma S, Voldman J (2018). Caring for cells in microsystems: principles and practices of cell-safe device design and operation. Lab Chip.

[56] Wang L-S, Boulaire J, Chan PPY, Chung JE, Kurisawa M (2010). The role of stiffness of gelatin-hydroxyphenylpropionic acid hydrogels formed by enzyme-mediated crosslinking on the differentiation of human mesenchymal stem cell. Biomaterials.

[57] Ross AM, Jiang Z, Bastmeyer M, Lahann J (2012). Physical aspects of cell culture substrates: topography, roughness, and elasticity. Small.

[58] van Delft FCMJM (2008). Manufacturing substrate nano-grooves for studying cell alignment and adhesion. Microelectron. Eng..

[59] Tran VD, Kumar S (2021). Transduction of cell and matrix geometric cues by the actin cytoskeleton. Curr. Opin. Cell Biol..

[60] Yang, W., Yu, H., Wang, Y. & Liu, L. Regulation of cell adhesion to poly(ethylene glycol) diacrylate film by modification with polystyrene nano-spheres. *In:* 2016 IEEE 16th International Conference on Nanotechnology (IEEE-NANO) 508–510 (2016). 10.1109/NANO.2016.7751442.

[61] Chauhan G (2020). Nano-spaced gold on glassy carbon substrate for controlling cell behavior. Adv. Mater. Interfaces.

[62] Ozbolat V (2018). 3D printing of PDMS improves its mechanical and cell adhesion properties. ACS Biomater. Sci. Eng..

[63] Gaio N (2016). Cytostretch, an organ-on-chip platform. Micromachines.

[64] Xu B (2018). Nanotopography-responsive myotube alignment and orientation as a sensitive phenotypic biomarker for duchenne muscular dystrophy. Biomaterials.

[65] Cassidy JW (2014). Osteogenic lineage restriction by osteoprogenitors cultured on nanometric grooved surfaces: the role of focal adhesion maturation. Acta Biomater..

[66] Lamers E (2010). The influence of nanoscale grooved substrates on osteoblast behavior and extracellular matrix deposition. Biomaterials.

[67] Kim MY, Li DJ, Pham LK, Wong BG, Hui EE (2014). Microfabrication of high-resolution porous membranes for cell culture. J. Memb. Sci..

[68] Pasman T, Grijpma D, Stamatialis D, Poot A (2018). Flat and microstructured polymeric membranes in organs-on-chips. J. R. Soc. Interface.

[69] Carter RN (2017). Ultrathin transparent membranes for cellular barrier and co-culture models. Biofabrication.

[70] Tibbe MP, Leferink AM, van den Berg A, Eijkel JCT, Segerink LI (2018). Microfluidic gel patterning method by use of a temporary membrane for organ-on-chip applications. Adv. Mater. Technol..

[71] Ashjari HR, Ahmadi A, Dorraji MSS (2018). Synthesis and employment of PEGDA for fabrication of superhydrophilic PVDF/PEGDA electrospun nanofibrous membranes by in-situ visible photopolymerization. Korean J. Chem. Eng..

[72] Liu J (2018). Pre-vascularization in fibrin Gel/PLGA microsphere scaffolds designed for bone regeneration. NPG Asia Mater..

[73] Haider A, Gupta KC, Kang I-K (2014). PLGA/nHA hybrid nanofiber scaffold as a nanocargo carrier of insulin for accelerating bone tissue regeneration. Nanoscale Res. Lett..

[74] Xu G (2017). Hyaluronic acid-functionalized electrospun PLGA nanofibers embedded in a microfluidic chip for cancer cell capture and culture. Biomater. Sci..

[75] Xue J, Wu T, Dai Y, Xia Y (2019). Electrospinning and electrospun nanofibers: methods, materials, and applications. Chem. Rev..

[76] Khorshidi S (2016). A review of key challenges of electrospun scaffolds for tissue-engineering applications. J. Tissue Eng. Regen. Med..

[77] Qin D, Xia Y, Whitesides GM (2010). Soft lithography for micro- and nanoscale patterning. Nat. Protoc..

[78] Tang, S. K. Y. & Whitesides, G. M. Basic Microfluidic and Soft Lithographic Techniques. *In:* Optofluidics: Fundamentals, Devices and Applications (eds. Fainman, Y., Lee, L. P., Psaltis, D. & Yang, C.) 7–31 (McGraw-Hill, 2010).

[79] Jang Y (2019). Comprehensive tuning of bioadhesive properties of polydimethylsiloxane ({PDMS}) membranes with controlled porosity. Biofabrication.

[80] Lamberti A, Marasso SL, Cocuzza M (2014). PDMS membranes with tunable gas permeability for microfluidic applications. RSC Adv..

[81] Mair DB (2022). PDMS-PEG block copolymer and pretreatment for arresting drug absorption in microphysiological devices. ACS Appl. Mater. Interfaces.

[82] Shirure VS, George SC (2017). Design considerations to minimize the impact of drug absorption in polymer-based organ-on-a-chip platforms. Lab Chip.

[83] Shakeri A, Khan S, Didar TF (2021). Conventional and emerging strategies for the fabrication and functionalization of PDMS-based microfluidic devices. Lab Chip.

[84] Cameron, T. C. et al. PDMS organ-on-chip design and fabrication: strategies for improving fluidic integration and chip robustness of rapidly prototyped microfluidic in vitro models. *Micromachines* **13**, 1573 (2022). 10.3390/mi13101573 PMC9609846 36295926

[85] Pocock KJ (2016). Low-temperature bonding process for the fabrication of hybrid glass-membrane organ-on-a-chip devices. J. Micro/Nanolithogr. MEMS.

[86] Zhou C, Ramiah Rajasekaran P, Wolff J, Li X, Kohli P (2010). Photo-pens: a simple and versatile tool for maskless photolithography. Langmuir.

[87] Hagedon M, Heikenfeld J (2013). A hybrid of microreplication and mask-less photolithography for creating dual porosity and textured surface membranes. J. Micromech. Microeng..

[88] Le-The H (2018). Large-scale fabrication of free-standing and sub-μm PDMS through-hole membranes. Nanoscale.

[89] Stucki AO (2015). A lung-on-a-chip array with an integrated bio-inspired respiration mechanism. Lab Chip.

[90] Shrestha J (2019). A rapidly prototyped lung-on-a-chip model using 3D-printed molds. Organs-on-a-Chip.

[91] Karlsson JM (2012). Fabrication and transfer of fragile 3D {PDMS} microstructures. J. Micromech. Microeng..

[92] Zhu D, Handschuh-Wang S, Zhou X (2017). Recent progress in fabrication and application of polydimethylsiloxane sponges. J. Mater. Chem. A.

[93] Wu M-H, Paul KE, Whitesides GM (2002). Patterning flood illumination with microlens arrays. Appl. Opt..

[94] Femmer T, Kuehne AJC, Wessling M (2014). Print your own membrane: direct rapid prototyping of polydimethylsiloxane. Lab Chip.

[95] Lei F (2021). Multi-compartment organ-on-a-chip based on electrospun nanofiber membrane as in vitro jaundice disease model. Adv. Fiber Mater..

[96] Qiu B (2022). Nanofiber self-consistent additive manufacturing process for 3D microfluidics. Microsyst. Nanoeng..

[97] Mashhadi Keshtiban, M., Moghimi Zand, M., Ebadi, A. & Azizi, Z. PDMS-based porous membrane for medical applications: Design, development, and fabrication. *Biomed. Mater*. 10.1088/1748-605X/acbddb. (2023). 10.1088/1748-605X/acbddb 36808922

[98] Ferreira DA (2021). A fast alternative to soft lithography for the fabrication of organ-on-a-chip elastomeric-based devices and microactuators. Adv. Sci..

[99] Schoen, F. J. & Mitchell, R. N. Chapter II.1.5 - Tissues, the Extracellular Matrix, and Cell–Biomaterial Interactions. in *Biomaterials Science* (Third Edition) (eds. Ratner, B. D., Hoffman, A. S., Schoen, F. J. & Lemons, J. E.) 452–474 (Academic Press, 2013). 10.1016/B978-0-08-087780-8.00039-5.

[100] Kubow KE (2015). Mechanical forces regulate the interactions of fibronectin and collagen I in extracellular matrix. Nat. Commun..

[101] Colombo E, Calcaterra F, Cappelletti M, Mavilio D, Della Bella S (2013). Comparison of fibronectin and collagen in supporting the isolation and expansion of endothelial progenitor cells from human adult peripheral blood. PLoS One.

[102] Sances S (2018). Human iPSC-derived endothelial cells and microengineered organ-chip enhance neuronal development. Stem Cell Rep..

[103] Kimura H, Yamamoto T, Sakai H, Sakai Y, Fujii T (2008). An integrated microfluidic system for long-term perfusion culture and on-line monitoring of intestinal tissue models. Lab Chip.

[104] Kimura H, Ikeda T, Nakayama H, Sakai Y, Fujii T (2015). An on-chip small intestine-liver model for pharmacokinetic studies. J. Lab. Autom..

[105] Zhang Y (2014). Disentangling the multifactorial contributions of fibronectin, collagen and cyclic strain on MMP expression and extracellular matrix remodeling by fibroblasts. Matrix Biol..

[106] Frantz C, Stewart KM, Weaver VM (2010). The extracellular matrix at a glance. J. Cell Sci..

[107] Pezzoli D (2018). Fibronectin promotes elastin deposition, elasticity and mechanical strength in cellularised collagen-based scaffolds. Biomaterials.

[108] Shah P (2016). A microfluidics-based in vitro model of the gastrointestinal human–microbe interface. Nat. Commun..

[109] Sajay, B. N. G., Yin, C. S. & Ramadan, Q. Optimization of micro-fabricated porous membranes for intestinal epithelial cell culture and in vitro modeling of the human intestinal barrier. *J. Micromech. Microeng.* **27**, 124004 (2017).

[110] Jang K-J (2013). Human kidney proximal tubule-on-a-chip for drug transport and nephrotoxicity assessment. Integr. Biol. (Camb.)..

[111] Sgarioto M (2012). Collagen type I together with fibronectin provide a better support for endothelialization. C. R. Biol..

[112] Sottile J (2007). Fibronectin-dependent collagen I deposition modulates the cell response to fibronectin. Am. J. Physiol. Cell Physiol..

[113] Urbanczyk M, Layland SL, Schenke-Layland K (2020). The role of extracellular matrix in biomechanics and its impact on bioengineering of cells and 3D tissues. Matrix Biol..

[114] Rens EG, Merks RMH (2020). Cell shape and durotaxis explained from cell-extracellular matrix forces and focal adhesion dynamics. iScience.

[115] Jang K-J, Suh K-Y (2010). A multi-layer microfluidic device for efficient culture and analysis of renal tubular cells. Lab Chip.

[116] Tan H-Y (2018). A multi-chamber microfluidic intestinal barrier model using Caco-2 cells for drug transport studies. PLoS One.

[117] van der Helm MW (2019). Non-invasive sensing of transepithelial barrier function and tissue differentiation in organs-on-chips using impedance spectroscopy. Lab Chip.

[118] Kasendra M (2018). Development of a primary human small intestine-on-a-chip using biopsy-derived organoids. Sci. Rep..

[119] Sontheimer-Phelps A (2020). Human colon-on-a-chip enables continuous in vitro analysis of colon mucus layer accumulation and physiology. Cell. Mol. Gastroenterol. Hepatol..

[120] Masuda H (2014). Coating extracellular matrix proteins on a (3-aminopropyl)triethoxysilane-treated glass substrate for improved cell culture. Biotechniques.

[121] Chiu J-J, Chien S (2011). Effects of disturbed flow on vascular endothelium: pathophysiological basis and clinical perspectives. Physiol. Rev..

[122] Snyder J (2020). Materials and microenvironments for engineering the intestinal epithelium. Ann. Biomed. Eng..

[123] Hughes CS, Postovit LM, Lajoie GA (2010). Matrigel: a complex protein mixture required for optimal growth of cell culture. Proteomics.

[124] Benam KH (2015). Engineered in vitro disease models. Annu. Rev. Pathol..

[125] García JR, Singh A, García AJ (2014). High fidelity nanopatterning of proteins onto well-defined surfaces through subtractive contact printing. Methods Cell Biol..

[126] Wright D (2008). Reusable, reversibly sealable parylene membranes for cell and protein patterning. J. Biomed. Mater. Res. A.

[127] Sergelen K, Petri C, Jonas U, Dostalek J (2017). Free-standing hydrogel-particle composite membrane with dynamically controlled permeability. Biointerphases.

[128] Hsieh H-Y (2014). Gradient static-strain stimulation in a microfluidic chip for 3D cellular alignment. Lab Chip.

[129] Zhang L (2015). Synthesis of pH-responsive hydrogel thin films grafted on PCL substrates for protein delivery. J. Mater. Chem. B.

[130] Yue K (2015). Synthesis, properties, and biomedical applications of gelatin methacryloyl (GelMA) hydrogels. Biomaterials.

[131] Sun, M. et al. Synthesis and properties of gelatin methacryloyl (GelMA) hydrogels and their recent applications in load-bearing tissue. *Polymers (Basel)*. **10**, 1290 (2018). 10.3390/polym10111290 PMC6401825 30961215

[132] Ye YN (2018). Tough and self-recoverable thin hydrogel membranes for biological applications. Adv. Funct. Mater..

[133] Yamato M (2007). Temperature-responsive cell culture surfaces for regenerative medicine with cell sheet engineering. Prog. Polym. Sci..

[134] Sun W, Hu Q, Ji W, Wright G, Gu Z (2017). Leveraging physiology for precision drug delivery. Physiol. Rev..

[135] Ratner, B.D., Hoffman, A.S., Schoen, F.J. & Lemons, J.E. Biomaterials science: an introduction to materials in medicine. *Chemical Engineering* (Academic Press, 2004).

[136] Wang C, Tanataweethum N, Karnik S, Bhushan A (2018). Novel microfluidic colon with an extracellular matrix membrane. ACS Biomater. Sci. Eng..

[137] Rayner SG (2018). Reconstructing the human renal vascular-tubular unit in vitro. Adv. Healthc. Mater..

[138] Hansen NUB, Genovese F, Leeming DJ, Karsdal MA (2015). The importance of extracellular matrix for cell function and in vivo likeness. Exp. Mol. Pathol..

[139] Jokhadar SZ, Suštar V, Svetina S, Batista U (2009). Time lapse monitoring of CaCo-2 cell shapes and shape dependence of the distribution of integrin β1 and F-actin on their basal membrane. Cell Commun. Adhes..

[140] Arık YB (2021). Collagen I based enzymatically degradable membranes for organ-on-a-chip barrier models. ACS Biomater. Sci. Eng..

[141] Sun Y-M (2014). In situ fabrication of a temperature- and ethanol-responsive smart membrane in a microchip. Lab Chip.

[142] Bakhchova, L. et al. Femtosecond laser-based integration of nano-membranes into organ-on-a-chip systems. *Materials (Basel)*. **13**, 3076 (2020). 10.3390/ma13143076 PMC7412128 32664211

[143] Herland A (2016). Distinct contributions of astrocytes and pericytes to neuroinflammation identified in a 3D human blood-brain barrier on a chip. PLoS One.

[144] Baeten KM, Akassoglou K (2011). Extracellular matrix and matrix receptors in blood-brain barrier formation and stroke. Dev. Neurobiol..

[145] Luo X (2010). In situ generation of pH gradients in microfluidic devices for biofabrication of freestanding{,} semi-permeable chitosan membranes. Lab Chip.

[146] Rosella E, Jia N, Mantovani D, Greener J (2021). A microfluidic approach for development of hybrid collagen-chitosan extracellular matrix-like membranes for on-chip cell cultures. J. Mater. Sci. Technol..

[147] Park G-S (2017). Emulating host-microbiome ecosystem of human gastrointestinal tract in vitro. Stem Cell Rev. Rep..

[148] Kim HJ, Ingber DE (2013). Gut-on-a-chip microenvironment induces human intestinal cells to undergo villus differentiation. Integr. Biol. (Camb.)..

[149] Wang T (2018). Layer-by-layer nanofiber-enabled engineering of biomimetic periosteum for bone repair and reconstruction. Biomaterials.

[150] Maschmeyer I (2015). Chip-based human liver-intestine and liver-skin co-cultures–a first step toward systemic repeated dose substance testing in vitro. Eur. J. Pharm. Biopharm..

[151] Bhattacharjee N, Urrios A, Kang S, Folch A (2016). The upcoming 3D-printing revolution in microfluidics. Lab Chip.

[152] Wolf MP, Salieb-Beugelaar GB, Hunziker P (2018). PDMS with designer functionalities—properties, modifications strategies, and applications. Prog. Polym. Sci..

[153] Huh D (2013). Microfabrication of human organs-on-chips. Nat. Protoc..

[154] Sackmann EK, Fulton AL, Beebe DJ (2014). The present and future role of microfluidics in biomedical research. Nature.

[155] Walker BW (2019). Engineering a naturally-derived adhesive and conductive cardiopatch. Biomaterials.

[156] Alizadeh R (2019). Conductive hydrogels based on agarose/alginate/chitosan for neural disorder therapy. Carbohydr. Polym..

[157] Zamprogno P (2021). Mechanical properties of soft biological membranes for organ-on-a-chip assessed by bulge test and AFM. ACS Biomater. Sci. Eng..

[158] Lesman A (2011). Engineering vessel-like networks within multicellular fibrin-based constructs. Biomaterials.

[159] Argentiere, S., Siciliano, P. A. & Blasi, L. How microgels can improve the impact of organ-on-chip and microfluidic devices for 3D culture: compartmentalization, single cell encapsulation and control on cell fate. *Polymers (Basel)*. **13**, 3216 (2021). 10.3390/polym13193216 PMC8512905 34641032

[160] Rahmani Dabbagh S (2023). 3D bioprinted organ-on-chips. Aggregate.

[161] Maurer M (2019). A three-dimensional immunocompetent intestine-on-chip model as in vitro platform for functional and microbial interaction studies. Biomaterials.

[162] van der Helm MW (2016). Direct quantification of transendothelial electrical resistance in organs-on-chips. Biosens. Bioelectron..

